# Controlling Photochromism of Donor‐Acceptor Stenhouse Adducts on Micro‐Dot Arrays Beyond Human‐Eyes Resolution for Dynamic Light Encryption

**DOI:** 10.1002/advs.75340

**Published:** 2026-05-07

**Authors:** Hongtao Hu, Fanxi Sun, Hanjun Zhang, Xiao Wang, Gaolu Zhu, Jiayu Li, Mengyao Yang, Xu Deng, Yonghao Zheng, Chen Wei, Dongsheng Wang

**Affiliations:** ^1^ Department of Pharmacy Sichuan Provincial People's Hospital School of Optoelectronic Science and Engineering University of Electronic Science and Technology of China Chengdu China; ^2^ School of Pharmacy Qujing University of Medicine & Health Sciences Qujing China; ^3^ Institute of Fundamental and Frontier Sciences University of Electronic Science and Technology of China Chengdu China

**Keywords:** donor‐acceptor stenhouse adducts, dynamic encryption, information security, photochromism

## Abstract

Conventional photoresponsive materials switch between two static states. Mastering the photoisomerization process expands their potential dynamic applications such as real‐time information displaying. Here we demonstrated precise control of the dynamics of solid‐state photochromism by introducing a “visual deception” strategy, where a micro‐dot array whose individual features are below the resolution limit of the human eyes. This array comprises photochromic and non‐photochromic pixels; the former are generated by inkjet‐depositing ester‐functionalized inks onto surfaces pretreated with donor‐acceptor Stenhouse adducts (DASAs). Under constant light irradiation, the photochromic kinetics is monotonically and uniformly increased with the increase of grayscale values, which is defined as the areal ratio of photochromic to total pixels. This fine‐tuned manipulation of photochromism enables the development of a dynamic light‐encryption technology, where fluorescent information is encoded at multiple grayscale levels using a high‐precision nanomaterial deposition inkjet printing system. The encrypted information is temporarily unveiled under steady light irradiation, appearing only within a specific time window. By introducing a temporal dimension to optical security, this technology establishes a new paradigm for advanced anti‐counterfeiting and information protection.

## Graphical abstract

This study presents a micro‐dot array strategy for dynamic light encryption, utilizing DASA‐based photochromism with resolution beyond human‐eye capacity. By precisely controlling isomerization kinetics, the method reveals multi‐layered encrypted information under specific light irradiation, enhancing information security and anti‐counterfeiting.

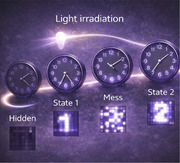

## 1 Introduction

**SCHEME 1:**
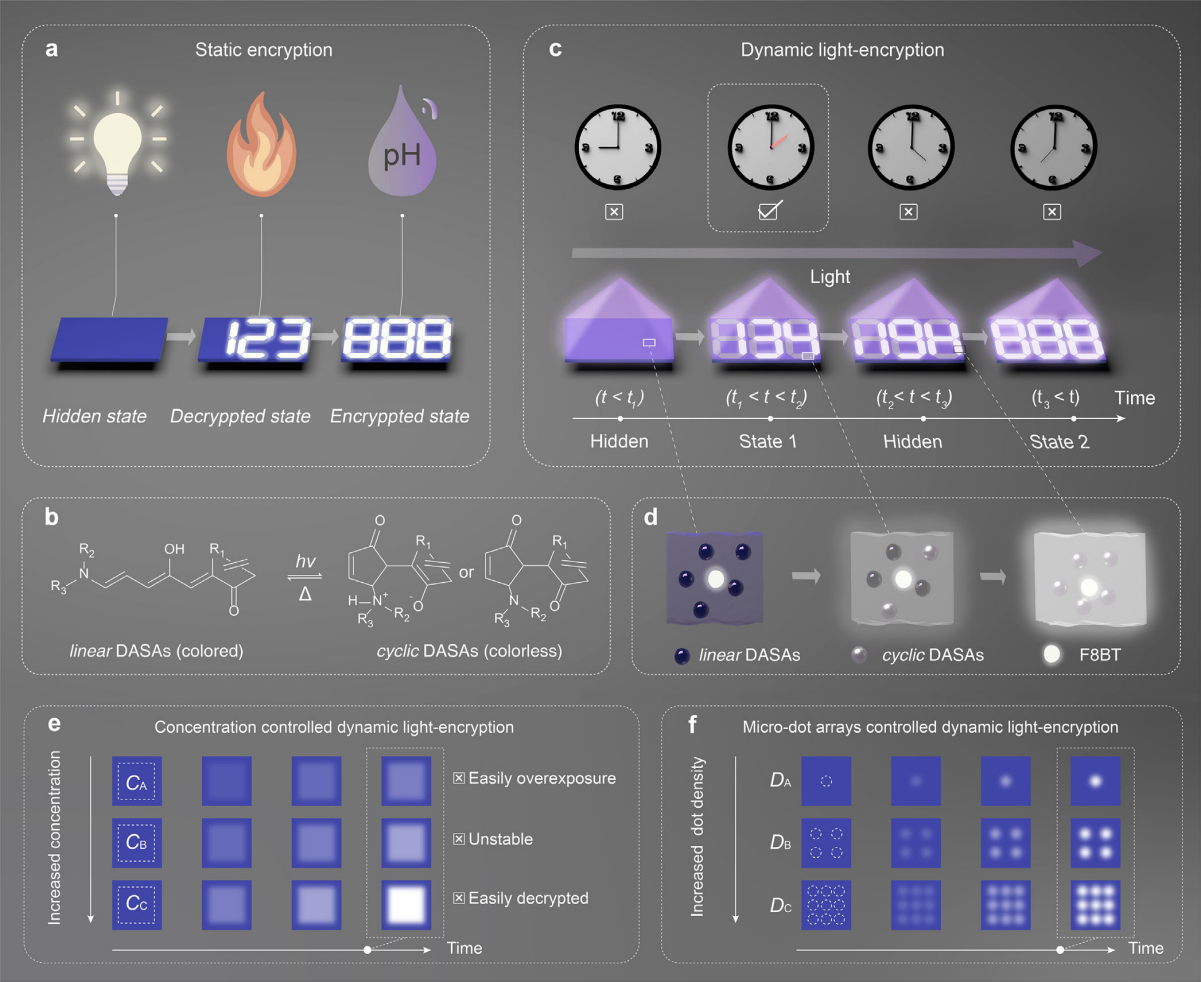
Mechanism of dynamic information encryption. (a) Schematic illustration of conventional static information encryption; (b) Schematic illustration of dynamic information encryption in the time dimension; (c) The chemical structure and corresponding photoisomerization of donor‐acceptor Stenhouse adducts (DASAs); (d) Schematic illustration of the *linear*‐to‐*cyclic* isomerization of DASAs gradually turning on the fluorescence of the underlying polymer; (e) Schematic illustration of concentration‐controlled dynamic light‐encryption, showing how varying concentrations (*C*_A_, *C*_B_, and *C*_B_) affect decryption process; (f) Schematic illustration of micro‐dot arrays controlled dynamic light‐encryption, illustrating the effect of varying dot densities (*D*_A_, *D*_B_, *D*_C_) on the decryption process.

Information security has long been a critical concern in military and intelligence operations, and remains equally vital today for safeguarding commercial secrets and combating counterfeiting of products, documents, and currency [1]. Most chromogenic materials are fundamentally limited by their binary color‐switching capability, enabling only static transitions between encrypted and decrypted states under specific external stimuli [2]. This simple switch‐type (on/off) control logic creates vulnerabilities to information leakage and unauthorized decryption [3]. Current encryption strategies primarily enhance security through two approaches (Scheme 1a): (1) Complicating decryption conditions by requiring specific sequences of multiple stimuli [4](e.g., light [5, 6, 7, 8, 9], heat [10], force [11], electricity [12], chemical agents [13, 14, 15]); (2) Increasing the number of decoy states that conceal the true information among multiple switchable states [2, 3]. These methods improve deceptiveness, making unauthorized interception difficult without knowledge of the precise decryption protocol.

However, most existing security technologies rely on static information display (Scheme 1a). In contrast, dynamic encryption offers a more advanced approach by embedding encrypted information within the flow of time, making it more difficult to intercept. Specifically, dynamic encryption enables the information to continuously change during the stimulation process, with different messages being revealed at various times (Scheme 1b). The introduction of temporal conditions into the encryption technology further improves the security of information. Dynamic encryption technology relies on the well‐controlling of kinetics of the decryption process [2, 9]. Photoresponsive molecules exhibit reversible time‐dependent isomerization between two stationary states under control of light irradiation, where the varying of chemical structure obviously switches the absorption spectra and color, making them attractive in developing the dynamic encryption technology [16, 17, 18, 19].

Donor‐acceptor Stenhouse adducts (DASAs) as typical T‐type photoresponsive molecules, exhibit visible‐light‐induced negative photochromism (*linear*‐to‐*cyclic* isomerization) and spontaneous thermal reversal (Scheme 1c) [20, 21, 22, 23]. Their dramatic absorbance changes in the visible region have been reported in fluorescence modulation through Förster resonance energy transfer (FRET) effect, where the *linear*‐to‐*cyclic* isomerization gradually turns on the fluorescence [24, 25]. Due to their push‐pull electronic structure, both the absorption spectra and isomerization kinetics of DASAs are strongly dependent on the chemical structure of the electron‐donating and ‐withdrawing moieties [26, 27, 28, 29]. However, well‐controlling photochromic kinetics in the solid state remains challenging, as the photoresponsive molecules spontaneously aggregate under high concentration, restricting the free volume required for isomerization as well as quenching the excited state [30, 31]. Our previous research demonstrated that ester‐functionalized molecules can significantly accelerate the *linear*‐to‐*cyclic* isomerization of DASAs in the solid state by facilitating the intramolecular proton transfer [32, 33, 34]. This approach holds great potential for precise kinetic control of solid‐state photochromism, which is essential for dynamic encryption yet remains largely unexplored.

In this work, we reported a dynamic light‐encryption technology for securing fluorescent information (Scheme 1b). Under continuous 420 nm light irradiation, the fluorescent information could be dynamically displayed on surface (Scheme 1d): (1) At the beginning stage, the emitted fluorescence is blocked by *linear* DASAs (encrypted state); (2) With prolonged exposure, the fluorescent‐induced *linear*‐to‐*cyclic* isomerization reduces visible‐light absorbance of DASAs, causing the information to appear gradually; (3) Crucially, distinct photochromic kinetics across different surface regions enable sequential information transformation over time.

Both our method and traditional approaches rely on controlling the kinetics to achieve multi‐layered information encryption. However, traditional methods typically rely on varying the “concentration” of the isomerization promoter [33]. Since the promoted regions are photoresponsive, the different concentrations lead to varying light sensitivity, often resulting in issues such as overexposure, uncontrollable kinetics, and spontaneous information leakage (Scheme 1e).

Uniquely, we achieve this by developing a “visual deception” strategy (Scheme 1f). A micro‐dot array with sub‐resolution features comprising both photochromic and non‐photochromic pixels is constructed. The photochromic pixels are created by inkjet‐printing ester‐functionalized inks. The photochromic kinetics are precisely tuned by the grayscale, which is defined as the ratio of photochromic to total pixel area (Scheme 1f). Using this approach, we demonstrate the dynamic display of encrypted fluorescent numeric and symbolic information. By introducing the temporal control, this method further improves the deceptiveness of information encryption, thereby demonstrating potential applications in the field of advanced information security and anti‐counterfeiting.

## 2 Results and Discussion

**FIGURE 1:**
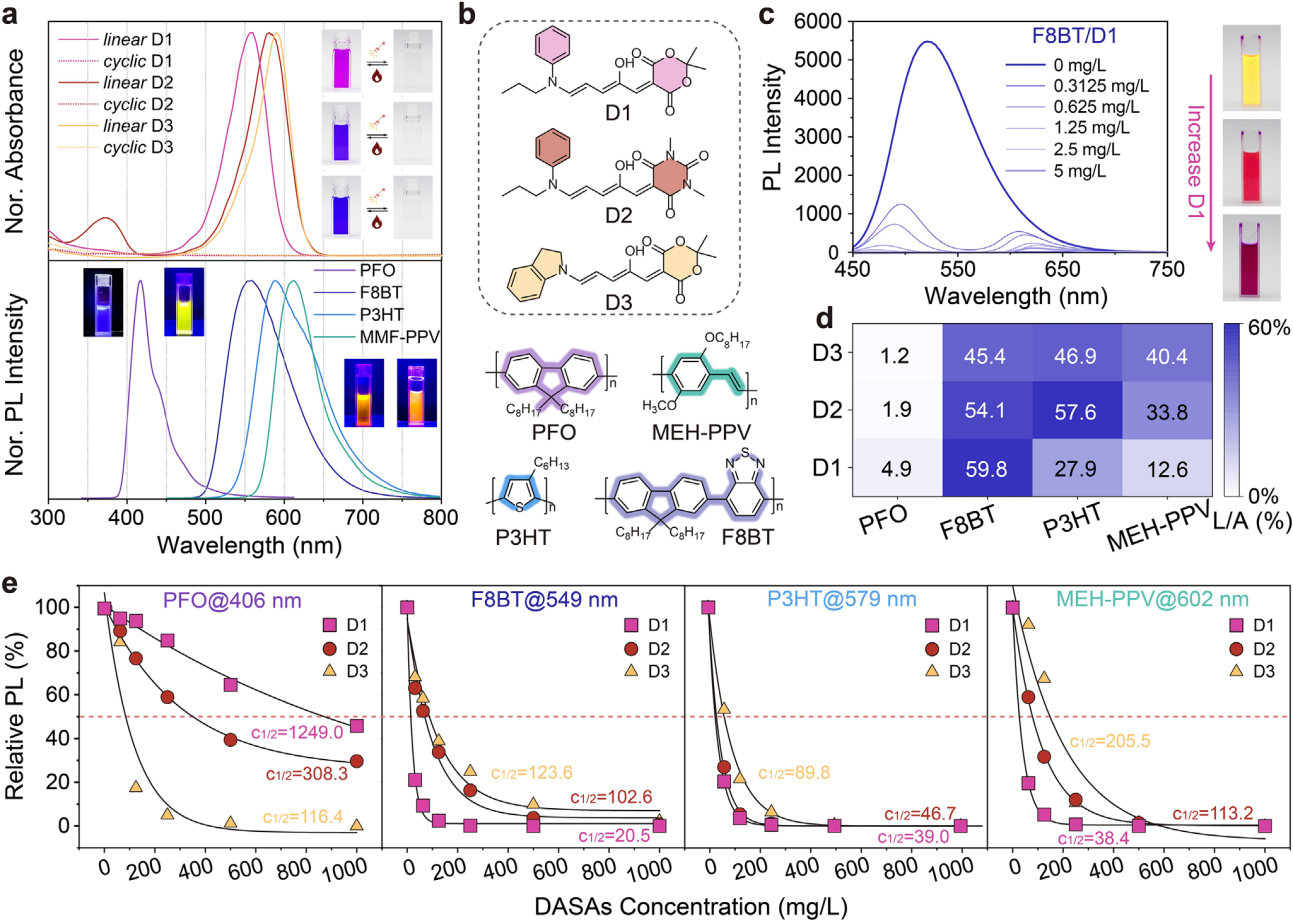
Photophysical characterization of DASAs and fluorescent polymers. (a) Normalized absorbance and fluorescence spectra of DASAs (*linear* and *cyclic* **D1**, **D2** and **D3** in dichloromethane (DCM)) and fluorescent polymers (**PFO**, **F8BT**, **PFO** and **P3HT** in DCM), insets show the photographic images of the corresponding solutions; (b) Chemical structures of the DASA photoswitches (**D1‐D3**) and fluorescent polymers; (c) Emission spectra of **F8BT** solutions (2.5 mg/L in DCM) upon successive addition of **D1**, photographic images reveal progressive fluorescence quenching; (d) Spectral overlap parameter (*R*%) between the emission band of each polymer and the absorbance band of each DASA photoswitch; (e) Plots of relative fluorescence intensity of polymer solutions (2.5 mg/L in DCM) as a function of added DASAs concentration (**D1‐D3**), probe wavelengths: **PFO**@406 nm, **F8BT**@549 nm, **MEH‐PPV**@579 nm, **P3HT**@602 nm.

A series of second generation of DASAs (**D1**‐**D3**) [29] were synthesized according to our previous reports [32, 33, 34], exhibiting narrow and sharp absorbance between ∼500 and ∼650 nm in dichloromethane (DCM), which generates bright and vivid colors spinning pink, purple and blue (Figure 1a,b). Visible light irradiation (520–590 nm) triggers the *linear*‐to‐*cyclic* isomerization, erasing the visible‐light‐region absorption and inducing a rapid colored‐to‐colorless transition (Figure 1a; Figure S1 and Tables S1 and S2). This typical negative photochromism arises from the decomposition of the triene *π*‐bridge and concomitant formation of the cyclopentenone ring, which disrupts the conjugation and push‐pull electronic structure [20, 21, 22].

Commonly used fluorescent polymers, including **PFO**, **F8BT**, **MEH‐PPV,** and **P3HT** were chosen because their emission maxima (406, 549, 579, and 602 nm, respectively) fall inside the absorption window of DASAs (Figure 1a,b) [20, 21, 22]. Gradual addition of DASAs into the solutions of fluorescent polymers attenuates and shifts the photoluminescence (Figure 1c; Figures S2–S5 and Table S3). This is attributed to that the whole or part of the fluorescence is absorbed by *linear* DASAs. The efficiency of this process is quantified by the spectral overlap parameter (*R*%):

(1) $$R\%\;=\frac{A_{i}}{A_{u}}\;\times 100\%$$

where *A_i_* and *A_u_* represent the area of intersection and union of the normalized emission and absorbance spectra, respectively (Figures S6–S8 and Table S4). The higher *R*% corresponds to stronger fluorescence re‐absorption and therefore to lower quencher concentrations required for encryption‐grade extinction (Figure 1d).

Because **PFO** emission lies almost entirely outside the DASAs absorption envelope (R < 5%), 50% attenuation (*c_1/2_*) for the fluorescence of **PFO** solution with the concentration of 2.5 mg/L requires > 4 mg/L D1, where the mass concentration ratio of **D1**/**PFO** is over ∼1.7 (Figure 1e; Figure S2). It is worth to note the *c_1/2_* is obtained by fitting the plots of relative fluorescent intensity for the polymer solutions under various concentration of DASAs with a one‐phase exponential decay function (Table S3). In contrast, **F8BT**, **MEH‐PPV,** and **P3HT** emit longer than 500 nm, consequently increases *R*% and decreases *c_1/2_* (Figure 1d,e). Typically, for the **D1**/**F8BT** pair (R = 59.8%), only ∼0.07 mg/mL **D1** is needed to halve the fluorescence, corresponding to a mass ratio of 0.03 and demonstrating an exceptionally high fluorescence‐blocking efficacy.

**FIGURE 2:**
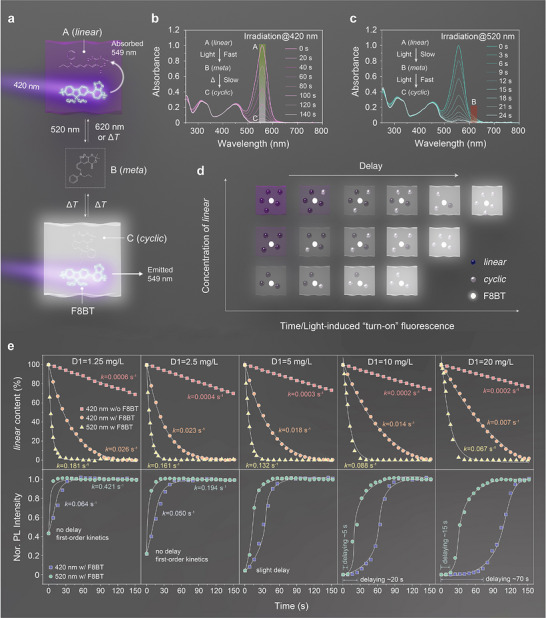
Light‐induced “turn‐on” fluorescence via isomerization. (a) Schematic illustration of the fluorescence‐triggered *linear*‐to‐*cyclic* photoisomerization mechanism of **D1**; (b) UV/vis traces (**D1** 5 mg/L + **F8BT** 2.5 mg/L in DCM) under 420 nm light irradiation (20 mW/cm^2^), the absorbance at 556 nm is normalized to 1, inset indicates the proposed A‐to‐C conversion pathway; (c) UV/vis traces (**D1** 5 mg/L + **F8BT** 2.5 mg/L in DCM) under 520 nm light irradiation (20 mW/cm^2^), the absorbance at 556 nm is normalized to 1, inset indicates the proposed A‐to‐C conversion pathway; (d) Concept: Schematic illustration of fluorescence‐induced *linear*‐to‐*cyclic* isomerization of DASAs which gradually turns on the fluorescence of the underlying polymer; (e) Time‐dependent plots for the residual *linear* **D1** fraction (upper) and normalized **F8BT** fluorescence (lower) under light irradiation. Conditions: 420 nm w/o **F8BT** (**D1** only, 20 mW/cm^2^ 420 nm light), 420 nm w/ **F8BT** (**D1**/**F8BT** mixed, 20 mW/cm^2^ 420 nm light), 520 nm w/ **F8BT** (**D1**/**F8BT** mixed, 20 mW/cm^2^ 520 nm light).

Unlike FRET‐based approaches, where the fluorescence of the donor molecule is used to excite the fluorescence of the acceptor molecule, relying on strong intermolecular dipole‐dipole coupling and distance‐dependent energy transfer, the fluorescence in our system is negligible from the DASAs. Instead, the fluorescence consistently originates from the fluorescent polymer. The generated fluorescence fulfils two indispensable roles in the dynamic light‐encryption technology: (1) it serves for the displaying of encrypted information; (2) it acts as an internal, self‐regulated light source to drive the *linear*‐to‐*cyclic* isomerization of DASAs (Figure 2a). A quantitative understanding of its temporal evolution is therefore mandatory. Owing to the well‐matched spectral overlap between the *linear* **D1** absorption and **F8BT** emission, **D1**/**F8BT** was selected as the photochromic/fluorophore pair for the subsequent studies.

Both 420 nm (violet) and 520 nm (green) light irradiation trigger the *linear*‐to‐*cyclic* isomerization of **D1** in the presence of **F8BT** (Figures S9 and S10). Interestingly, the spectral evolutionary process is different for the two pathways: while 420 nm light induces a slow but monotonic decrease of the absorbance in the visible light region, 520 nm light generates a small absorption band between ∼600 and ∼650 nm at the beginning stage, which quickly disappears with the proceeding of photoisomerization (Figure 2b,c). Generally, the *linear*‐to‐*cyclic* isomerization of DASAs includes a complex molecular transition process with 15 intermediates [35, 36, 37]. This absorption band above ∼600 nm corresponds to the formation of intermediate B, which is an important and metastable species that precedes cyclization (Figure 2a–c). The A (*linear*)‐to‐B transformation is initiated by the n‐π* excitation of the triene *π*‐bridge, following by the sequential *Z/E* inversion of the C═C bonds. The intermediate B is unstable and switches to C (*cyclic*) through a thermal relaxation process (Figure 2a). With 420 nm illumination the broad **F8BT** fluorescence (∼500–700 nm) simultaneously promotes both the A‐to‐B (dominated) and B‐to‐A transition, preventing accumulation of intermediate B (Figure 1a,b). In contrast, the narrow 520 nm line preferentially accumulates intermediate B, making it experimentally detectable (Figure 2c).

Kinetic analysis reveals strictly first‐order behavior for the *linear*‐to‐*cyclic* isomerization under both 420 and 520 nm light irradiation, implying that external illumination and **F8BT**‐generated fluorescence act equivalently on the isomerization (Figure 2d,e; Table S5). In the absence of **F8BT**, <25% of *linear* **D1** switches to *cyclic* after 150 s of 420 nm exposure, reflecting the weak tail of the light‐emitting diode (LED) emission spectrum (Figure 2e; Figures S11 and S12). The rate constant (*k*) is obtained by fitting the plots with a first‐order kinetics curve (see details in SI). Compared with 520 nm light irradiation, the fluorescence‐induced *linear*‐to‐*cyclic* isomerization exhibits much lower rate constants, which are monotonically decreased from 0.026 to 0.007 s^−1^ as the **D1** concentrations increase from 1.25 to 20 mg/L. These might be on the one hand attributed to aggregation‐induced self‐quenching under high concentrations [38, 39]; on the other hand, the efficiency of fluorescence for triggering the isomerization is concomitantly decreased.

The *linear*‐to‐*cyclic* isomerization of **D1** gradually turns on the fluorescence because of the sharply decreased absorbance in the visible light region (Figures S13 and S14). Interestingly, the turning‐on dynamics is decoupled from the isomerization, which could be programmed by simply tunning the **D1**/**F8BT** weight ratios (Figure 2d,e).

1. Dilute regime ([**D1**] < 2.5 mg/L, **D1**:**F8BT** < 1:1). The absorbance of *linear* **D1** is insufficient to completely quench **F8BT**, so the fluorescence rises immediately upon illumination. The turning‐on process follows typical first‐order kinetics (Figure 2e; Table S6). The rate constants keep decreasing with the increased **D1** concentrations, which is in good accordance with the results of isomerization.
2. Concentrated regime ([**D1**] = 5 mg/L, **D1**:**F8BT** > 2:1). The fluorescence is completely quenched at initial. Fluorescence remains off until a critical fraction of **D1** has isomerized, which results in a delay of the turning‐on process. The delaying time lengthens linearly with the **D1** concentrations, reaching ∼70 s at 20 mg/L (**D1**:**F8BT** = 8:1). Excitation at 520 nm shortens the delay because of the faster isomerization at this wavelength.

This concentration‐gated, wavelength‐selective control of the fluorescence turn‐on furnishes a convenient handle for programming temporal encryption keys in dynamic light‐encoded security labels.

**FIGURE 3:**
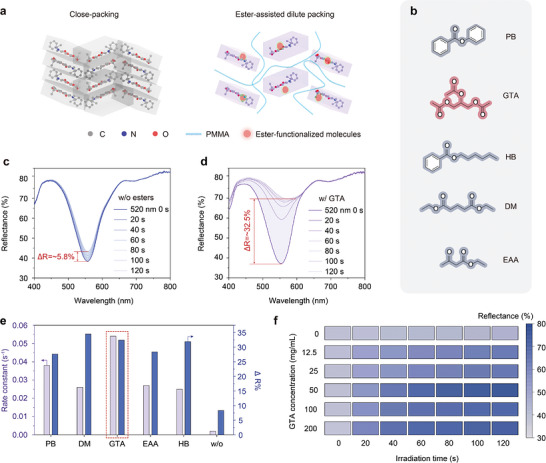
Ester‐assisted solid‐state photochromism. (a) Schematic illustration of ester‐promoted photoisomerization of solid‐state DASAs; (b) The chemical structures of the following five esters: phenyl benzoate (**PB**), glycerol triacetate (**GTA**), hexyl benzoate (**HB**), diethyl malonate (**DM**), and ethyl acetoacetate (**EAA**); (c) Diffuse‐reflectance spectra of photographic paper treated by **D1** (10 mg/mL in DCM, without esters) under 520 nm light irradiation (20 mW/cm^2^); (d) Diffuse‐reflectance spectra of photographic paper treated by **D1** (10 mg/mL in DCM, with 200 mg/mL GTA) under 520 nm light irradiation (20 mW/cm^2^); (e) Rate constants and Δ*R*% for DASA isomerization accelerated by five types of ester groups versus DASA isomerization without ester‐group acceleration; (f) Mapping of reflectance values of photographic paper treated by **D1** (10 mg/mL in DCM, with various concentrations of **GTA**) under 520 nm light irradiation (20 mW/cm^2^).

Photoresponsive molecules often exhibit strong intermolecular aggregation in the solid state, which is attributed to the planar and conjugated molecular nature (Figure 3a) [40, 41]. These limit the necessary free space required for photoisomerization. In our previous research, we demonstrated that ester‐functionalized compounds dramatically facilitate the *linear*‐to‐*cyclic* isomerization of DASAs in the solid state by on the one hand optimizing molecular packing and on the other hand, promoting the intramolecular proton transfer from the hydroxyl on triene *π*‐bridge to the carbonyl on electron‐withdrawing moiety, thereby stabilizing the *cyclic* isomers [32, 33, 34]. To investigate this effect, **D1** (10 mg/mL) was co‐deposited with various ester‐functionalized compounds including phenyl benzoate (**PB**), hexyl benzoate (**HB**), diethyl malonate (**DM**), glyceryl triacetate (**GTA**), and ethyl acetoacetate (**EAA**) (each at 200 mg/mL) onto glossy photographic paper via solution soaking in DCM (Figure 3b; Figure S15). Trace amounts of polymethyl methacrylate (PMMA, 1 mg/mL) were incorporated to dilute molecular packing and prevent undesired color shifts induced by the ester additives (Figure 3a). The photochromic behavior was investigated by monitoring the reflectance spectra in the visible region. In the absence of ester additives, **D1** in the solid state exhibits negligible *linear*‐to‐*cyclic* isomerization under 520 nm light irradiation, consistent with our previous results (Figure 3c) [34]. All the ester‐functionalized compounds markedly promoted the *linear*‐to‐*cyclic* isomerization (Figure 3d; Figures S16–S19). Notably, with **GTA**, **D1** exhibits a ∼32.4% increase in the reflectance at 556 nm at photoequilibrium with a rate constant of ∼0.055 s^−1^, approaching the values observed in solution (Figure 3e; Figure S20 and Table S7).

The promoting effect to the isomerization by ester concentration was quantitatively investigated by depositing GTA solutions with various concentrations onto **D1**‐impregnated photographic paper surface (see details in SI). Both the isomerization kinetics and photoequilibrium conversion efficiency increased progressively with **GTA** concentration (Figures S21–S26). Consequently, at fixed irradiation times, **D1** exhibits the **GTA**‐concentration‐dependent reflectance values (Figure 3f; Figures S27 and S28). These have been applied in the encryption of grayscale information [33, 34]. Nevertheless, the relationship between reflectance and **GTA** concentration is non‐linear at all irradiation times, complicating precise control over photochromic dynamics (Table S8). Thus, achieving robust control over solid‐state photochromism of DASAs remains a significant challenge.

**FIGURE 4:**
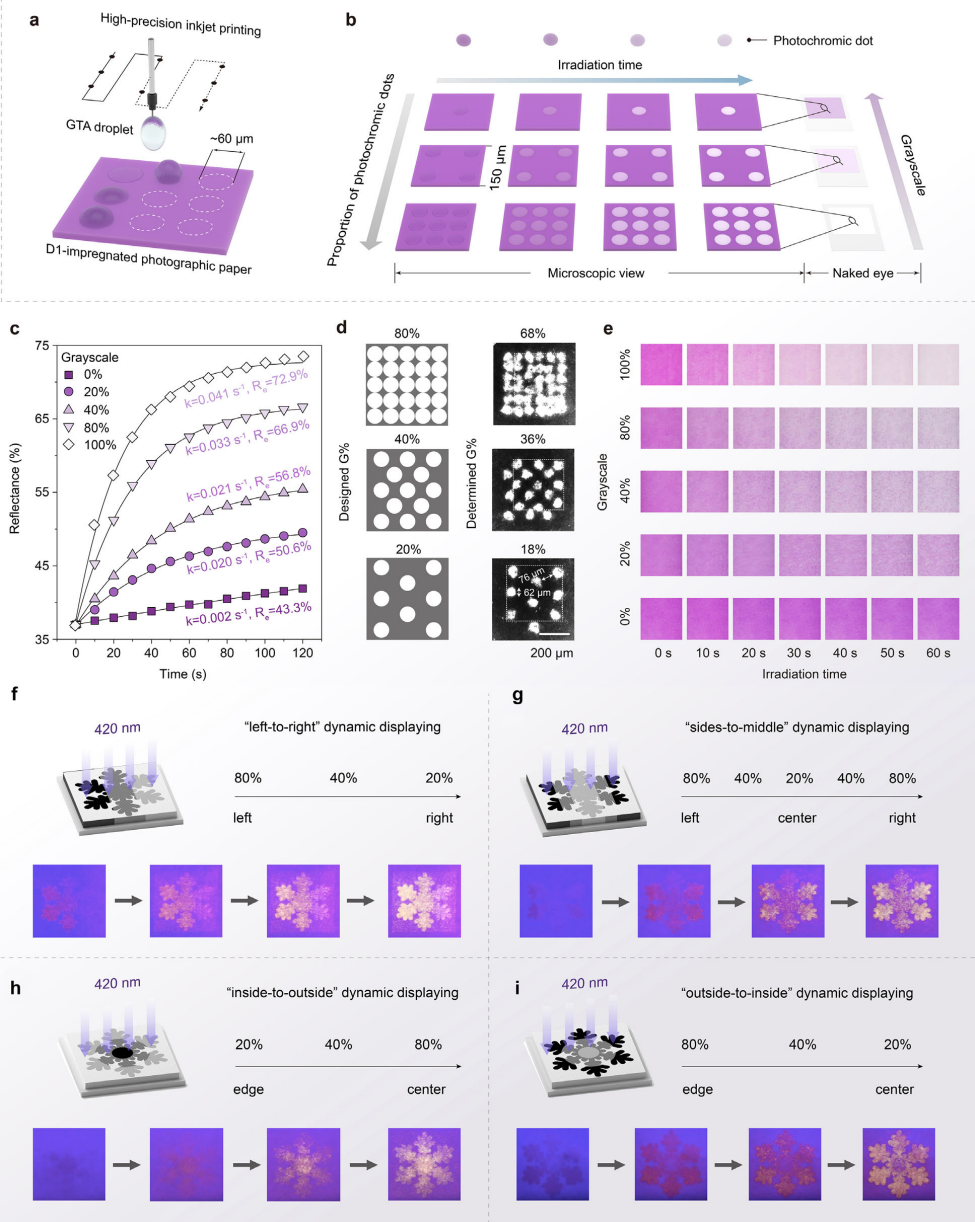
Dynamic display of fluorescent patterns on the surface. (a) Schematic illustration of the working mechanism of high‐precision inkjet printing; (b) Schematic illustration of how different dot matrices form various macroscopic grayscale levels on photographic paper under the visual resolution limit of the human eye; (c) Reflection kinetic curves of dot matrices with different grayscale levels: 0%, 20%, 40%, 80%, and 100%; (d) Schematic illustrations and optical microscope images of dot matrices with 20%, 40%, and 80% grayscale level; (e) Actual photographs of 0%, 20%, 40%, 80%, and 100% grayscale patterns on photographic paper under 520 nm light illumination (20 mW/cm^2^); (f) Snowflake patterns with varying grayscale gradients printed on photographic paper, displayed from left to right under 420 nm light illumination (20 mW/cm^2^); (g) Snowflake patterns with varying grayscale gradients printed on photographic paper, displayed from sides‐to‐middle under 420 nm light illumination (20 mW/cm^2^); (h) Snowflake patterns with varying grayscale gradients printed on photographic paper, displayed from inside to outside under 420 nm light illumination (20 mW/cm^2^); (i) Snowflake patterns with varying grayscale gradients printed on photographic paper, displayed from outside to inside under 420 nm light illumination (20 mW/cm^2^).

The human eyes typically exhibit a minimum resolvable angle of 1 arcminute [42]. That means at a viewing distance of 50 cm, this corresponds to a resolution limit of approximately 150 µm, rendering dots with smaller separations indistinguishable and perceived as uniform grayscale (Figure 4a,b). The grayscale values monotonically and linearly increase with the areal proportion of black dots. Inspired by this principle, we developed a “visual deception” strategy to precisely control the kinetics of DASAs photochromism on the surface. A micro‐dot array was constructed with photochromic and non‐photochromic pixels (Figure 4b). The photochromic dots were generated by inkjet‐depositing ester‐functionalized inks onto **D1**‐pretreated surface, which was realized using a high‐precision nanomaterial deposition inkjet printing system (Figure 4a). The ester‐functionalized inks formulation comprised **GTA** (200 mg/mL) dissolved in ethanol, delivered through a piezoelectric nozzle (see details in Supporting Information). The printing position is under precise control of a 3‐axis motion stage with 1 µm bi‐directional repeatability, ensuring all the features remain below the human visual resolution limit.

The grayscale values (*G*%) were quantified as the percentage of area covered by photochromic pixels:

(2) $$G\%\;=\frac{A_{p}}{A_{p}+A_{n}}\;\times 100\%$$

where *A_p_* and *A_n_* denote the total areas of photochromic and non‐photochromic pixels, respectively. Since *A_p_* = *n_p_* × *A*_0_  (*n_p_* the number of photochromic pixels; *A*_0_ the area of a single pixel), the pixel size is critical and determined by both the nozzle diameter and ink surface tension. Statistical analysis across multiple prints yielded an average pixel diameter of ∼60 µm (Figures S29 and S30).

Micro‐dot arrays with designed grayscale values spinning 0%, 20%, 40%, 80%, and 100% were fabricated (Figure 4c,d). To calibrate grayscale accuracy, control samples were prepared using **D1** solution (10 mg/mL in ethanol) as a tracer. (Figure 4d) The grayscale values 0% and 100% were achieved by soaking the photographic paper into the ethanol without and with **D1**, respectively. Measured grayscale values were 0%, 18%, 36%, 70%, and 100%, showing minor negative deviation from the design specifications. Importantly, inter‐pixel spacing in all arrays remained below the 150 µm resolution threshold at the 50 cm viewing distance (Figure 4d).

Under 520 nm green light irradiation, the *linear*‐to‐*cyclic* isomerization of **D1** progressively increases the reflectance in the visible light region, thus resulting in a color transition from pink to white (Figure 4c; Figures S31–S35). Both the kinetics and the equilibrium conversion rate of this isomerization increase with higher grayscale values (Table S9). Importantly, compared to variations in the GTA concentration, the kinetic curves for the photochromism of **D1** on the surface are uniformly distributed across the grayscale values. This uniformity enables precise control over reflectance and color at any given irradiation time (Figure 4c,e). Furthermore, the dependence of reflectance on irradiation duration is predictable. The photochromism of **D1** arises from the integrated response of the photochromic and non‐photochromic dots, which could be quantified using the following equation (Figure S36, see detain in SI):

(3) $$R_{G}=G\%\times R_{1}+\left(1-G\%\right)\times R_{0}$$

where *R_G_*, *R*_1_ and *R*_0_ represent the reflectance values at 556 nm at specific irradiation time, corresponding to the grayscale of *G*%, 100%, and 0%, respectively. This model guarantees accurate design and regulation of color evolution at different surface positions under light irradiation (Figure S38).

The precise control over **D1**’s photochromism enables dynamic display of fluorescent information on surfaces under continuous light irradiation. We fabricated snowflake patterns featuring various grayscale value gradients (i.e., left‐to‐right, inside‐to‐outside, outside‐to‐inside, and sides‐to‐middle) on the photographic paper using the high‐precision nanomaterial deposition inkjet printing system (Figure 4a,f–i). The photographic paper was pretreated with a mixed solution of **D1**, **F8BT,** and PMMA (10, 5, and 1 mg/mL in DCM).

Grayscale value gradients were generated by printing **GTA** (200 mg/mL in ethanol) at grayscale values of 1%, 20%, 40% and 80% in specific sequences. Notably, the grayscale value of 1% served as a background to prevent the piezoelectric nozzle clogging. Taking the “left‐to‐right” sequence as an example, the snowflake pattern was divided into three sections with grayscale values of 80%, 40%, and 20% from left to right (Figure 4f). Irradiation at 420 nm progressively turns on the **F8BT** fluorescence, resulting in a “left‐to‐right” emergence of the fluorescent pattern (Figure 4f; Movie S1). Similar approaches enabled “inside‐to‐outside”, “outside‐to‐inside” and “sides‐to‐middle” sequences (Figure 4g–i; Movies S2–S4). These demonstrate that temporal control in fluorescent information display can be introduced by engineering photochromic kinetics gradients on surfaces. Important, the dynamic display sequence can be precisely predetermined.

**FIGURE 5:**
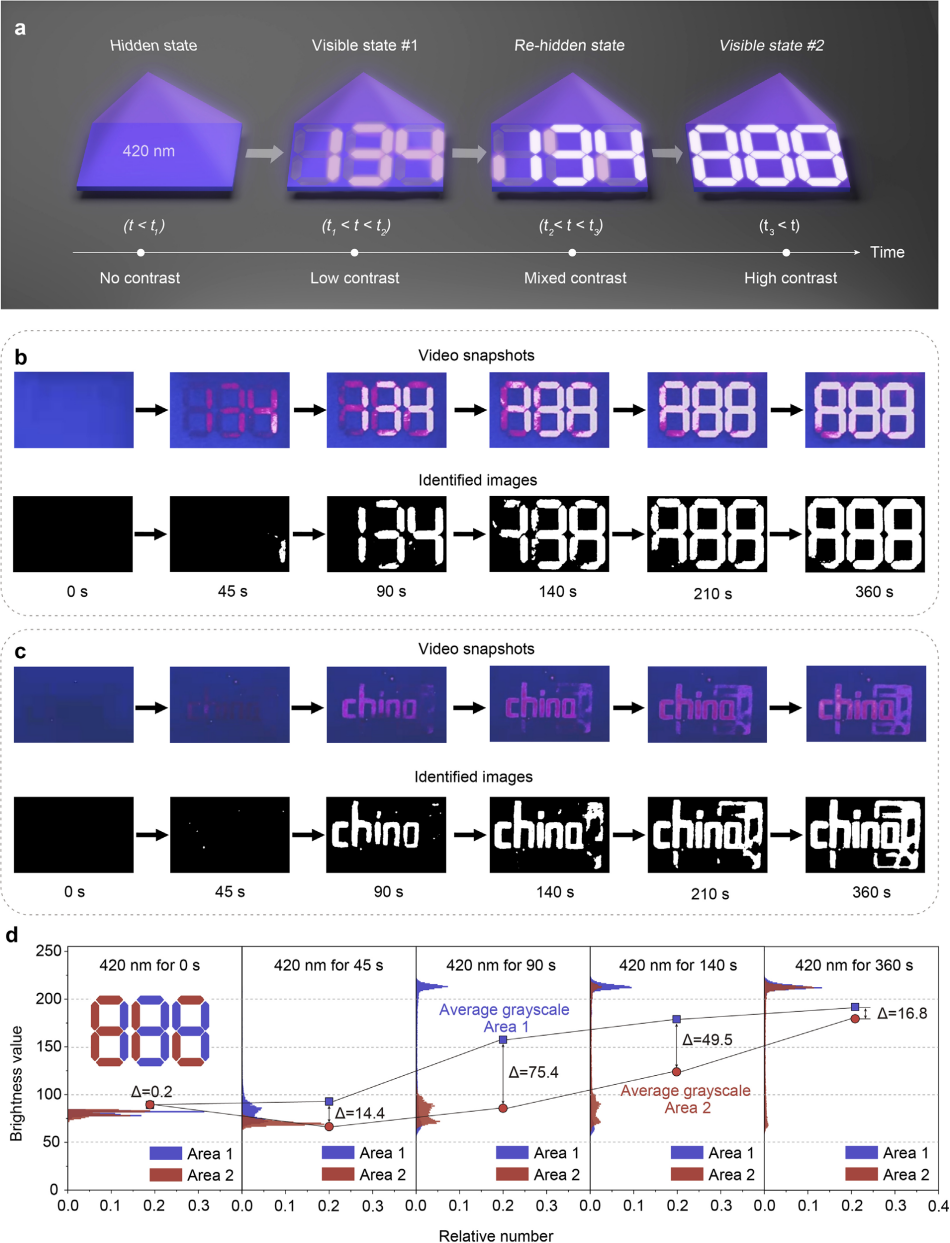
Dynamic light‐encryption of fluorescent information. (a) Schematic illustration of numeric patterns consisted of grayscale gradients exhibits sequential emergence under continous light irradiation on surface; (b) Video snapshots of the numeric pattern under 420 nm light irradiation (20 mW/cm^2^), with the identified images showing sequential emergence of “134” and “888”; (c) Video snapshots of the symbolic pattern under 420 nm light irradiation (20 mW/cm^2^), with the identified images showing sequential emergence of “China” in English and Chinese; (d) Time‐dependent brightness distribution across different regions of the numeric pattern under 420 nm light irradiation (20 mW/cm^2^).

A dynamic light‐encryption technology was further developed and applied for the secrecy and anti‐counterfeiting of fluorescent information (Figure 5a). Numeric and symbolic patterns were printed on the photographic paper surface using GTA inks (200 mg/mL in ethanol) with grayscale values of 1%, 40%, and 80% (Figure S37). The background was printed with a grayscale of 1% to prevent nozzle clogging. The digits “134” were printed at a grayscale of 80% and the remaining area at 40%. Under 420 nm light irradiation, the fluorescent visible state #1 “134” gradually emerged (t = 0–90 s), follewed by a re‐hidden state (t = 140–210 s), then transitioned to visible state #2 “888” upon prolonged exposure (t = 90–360 s) (Figure 5a,b; Movie S5). For the symbolic pattern, the English and Chinese characters “China” were printed with the grayscale of 80% and 40%, respectively (Figure S37). Light irradiation induces the appearance of the English word first, followed by the Chinese word to form a complete pattern (Figure 5c; Movie S6).

A self‐written brightness‐analysis software was employed to quantitatively evaluate the encryption performance (source code available in Section S6). The brightness evolution over irradiation time across spatial regions was analyzed for the numeric pattern (Figure 5d). During initial irradiation, area 1 and 2 exhibited similar average brightness values (∼75) with negligible difference (∼0.2), rendering them indistinguishable to both visual inspection and software analysis. Prolonged irradiation sharply increased the brightness of area 1, while the brightness of area 2 remained relatively low (Figure 5b,d). At an irradiation time of 90 s, the average brightness difference between the two areas reached ∼75.4, revealing the encrypted information. Further irradiation to 360 s increased brightness in area 2, reducing the difference to ∼16.8 and re‐concealing the information. These results demonstrate that dynamic light‐encryption can confine fluorescent information display to a specific time window, thereby enhancing information security.

## 3 Conclusions

In summary, we developed a “visual deception” strategy for precisely controlling the solid‐state photochromic dynamics of DASAs by constructing a micro‐dot array comprising photochromic and non‐photochromic pixels beyond human‐eyes resolution. The kinetics of photochromism on the surface monotonically increase with the grayscale values, defined as the ratio of photochromic area to total area. A dynamic display of fluorescent information was achieved on surfaces, which further guided the development of a dynamic light‐encryption technology for secrecy and anti‐counterfeiting. Under continuous irradiation, the fluorescent information gradually appears and subsequently re‐hides with extended exposure. By introducing temporal constraints, this technology confines information appearance to a specific time window, thereby enhancing encryption security.

Conventional photochromism control relies on homogeneous tuning of physicochemical properties. In contrast, this work presents a strategy for constructing surfaces with discontinuous, binary‐patched topographies beyond human‐eye resolution, enabling precise kinetic control through binary‐area modulation. We envision that this approach will extend to regulating chemical reactions, material properties, and biological processes, thereby advancing the design and manipulation of stimuli‐responsive materials and devices.

## Funding

This research was funded by the National Natural Science Foundation of China (22375029 and 52203134) and the Foundation of Science & Technology Department of Sichuan Province (2023ZYD0037, 2024YFHZ0307, and 2024NSFSC0249).

## Conflicts of Interest

The authors declare no conflicts of interest.

## Supporting information




**Supporting File 1**: advs75340‐sup‐0001‐SuppMat.docx.

**Supporting File 2**: advs75340‐sup‐0002‐MovieS1‐S6.zip.

## Data Availability

The data that support the findings of this study are available from the corresponding author upon reasonable request.
