# Supplementary material for: Controlling Photochromism of Donor‐Acceptor Stenhouse Adducts on Micro‐Dot Arrays Beyond Human‐Eyes Resolution for Dynamic Light Encryption
Source: Adv Sci (Weinh). 2026 May 7;13(38):e75340. doi: 10.1002/advs.75340 (PMC13335455; doi:10.1002/advs.75340)
Supplement: Supplementary file 1 — Supporting File 1: advs75340‐sup‐0001‐SuppMat.docx. [file ADVS-13-e75340-s001.docx]

Supporting Information

**Controlling photochromism of donor-acceptor Stenhouse adducts on micro-dot arrays beyond human-eyes resolution for dynamic light encryption**

*Hongtao Hu, Fanxi Sun*, Hanjun Zhang*, Xiao Wang, Gaolu Zhu, Jiayu Li, Mengyao Yang, Xu Deng, Yonghao Zheng*,* *Chen Wei, Dongsheng Wang**

H. Hu, F. Sun*, H. Zhang*, X. Wang, G. Zhu, J. Li, M. Yang, Y. Zheng*, C. Wei, D. Wang*

Department of Pharmacy, Sichuan Provincial People’s Hospital, School of Optoelectronic Science and Engineering, University of Electronic Science and Technology of China, Chengdu, China

*Corresponding authors. Email: [fanxi_sun@uestc.edu.cn](mailto:fanxi_sun@uestc.edu.cn) (F. S.); [hanjunzhang@qjmc.edu.cn](mailto:hanjunzhang@qjmc.edu.cn) (H. Z.) [zhengyonghao@uestc.edu.cn](mailto:zhengyonghao@uestc.edu.cn) (Y. Z.); [wangds@uestc.edu.cn](mailto:wangds@uestc.edu.cn) (D. W.)

H. Zhang

School of Pharmacy, Qujing University of Medicine & Health Sciences, Qujing 655100, China.

X. Deng

Institute of Fundamental and Frontier Sciences, University of Electronic Science and Technology of China, Chengdu, China

**Table of Contents**

1 Supporting Methods 3

1.1 Materials 3

1.2 Characterization 3

1.3 Synthesis 4

2 Photophysical properties of DASAs and fluorescent polymers 7

2.1 Photoisomerization of DASAs 7

2.2 Fluorescence re-absorption by DASAs 8

2.3 Overlapping between the absorption and emission spectra 10

3 Photodynamic properties in solutions 13

3.1 Dynamics of linear-to-cyclic isomerization of DASAs 13

3.2 in-situ emission spectra of D1/F8BT solutions 18

4 Photoisomerization dynamic properties in solid 21

4.1 Deposition of ester-functionalized compounds on surface 21

4.2 Photochromism dynamics promoted by ester-functionalized compounds 22

4.3 dynamics promoted by GTA with various concentrations 25

4.4 Photoisomerization dynamics controlled by grayscale 30

5 Supplementary movies 39

6 Source code 40

7 Supporting References 42

# Supporting Methods

## Materials

All the chemicals and reagents were used without further purification. F8BT (CAS No. 210347-52-7) was purchased from Yuanye Bio-Technology. Poly(methyl methacrylate) (CAS No. 9011-14-7), glyceryl triacetate (C_9_H_14_O_6_, CAS No. 102-76-1), 1,3-dimethylbarbituric acid (C_6_H_8_N_2_O_3_, CAS No. 769-42-6) were purchased from Aladdin. Poly(2-methoxy-5-(2-ethylhexyloxy)-1,4-phenylenevinylene) (CAS No. 138184-36-8), 2,2-dimethyl-1,3-dioxane-4,6-dione (C_6_H_8_O_4_, CAS No. 2033-24-1), 2-furaldehyde (C_5_H_4_O_2_, CAS No. 98-01-1) were purchased from Meryer. Poly(3-hexylthiophene-2,5-diyl) (CAS No. 104934-50-1) was purchased from Macklin. Indoline (C_8_H_9_N, CAS No. 496-15-1) was purchased from Tianjin HOPE. Poly((9,9-dioctylfluorenyl-2,7-diyl)-alt-(2,6-pyridine)) (CAS No. 773895-96-8) was purchased from Derthon. N-Propylaniline (C_9_H_13_N, CAS No. 622-80-0) was purchased from Shanghai Titan. Sodium bisulfite (HNaO_3_S, CAS No. 7631-90-5) was purchased from anpel. Dichloromethane (DCM) (CH_2_Cl_2_, CAS No. 75-09-2), n-Hexane (C_6_H_14_, CAS No. 110-54-3), ethyl acetate (EA) (C_4_H_8_O_2_, CAS No. 141-78-6), ethanol (EtOH) (C_2_H_6_O, CAS No. 64-17-5) were purchased from General-Reagent. Milli-Q water (resistivity: 18.2 MΩ×cm) was used throughout the project.

## Characterization

All spectra were measured at room temperature (RT: 23-25 ^o^C). Nuclear magnetic resonance (NMR) spectra were collected on a Bruker AVANCE III HD 400 MHz spectrometer to analyze molecular structures. Matrix-assisted laser desorption/ionization time of flight mass spectra (MALDITOF-MS) were measured on a Shimazu AXIMA Performance.

UV/vis spectra (absorption, diffuse reflectance) were recorded on a Shimadzu UV-2600 spectrophotometer and a Shimadzu Lightway PQY-01. Fluorescent emission spectra were measured on a Hitachi F-4600 Fluorescence Spectrophotometer (static test) and a home-made *in-situ* fluorescence measurement system (dynamic test).

The light-emitting diodes (LEDs) of violet light (420 nm) and green light (520 nm) were used to trigger polymer fluorescence and induce photoisomerization of donor-acceptor Stenhouse adducts (DASAs). The emission spectra of LED were obtained through a SpectraScan PR-655-Photo Research. The output intensity of the LEDs was controlled by a controller (Zhongjiao Jinyuan Systems).

Ink printing was performed with a MicroFab JetLab 4 (RUIDU). The droplet diameter is approximately 30 μm, with a minimum spacing of about 10 μm between droplets.

## Synthesis

All the DASAs were synthesized according to a modified strategy based on the previous reports.^[1]^

***(E)-5-(furan-2-ylmethylene)-1,3-dimethyl-6-methylenedihydropyrimidine-2,4(1H, 3H)-dione (S1)***

2,2-Dimethyl-1,3-dioxane-4,6-dione (1.44 g, 10 mmol) was dissolved into 50 mL distilled water under stirring. 2-Furancarboxaldehyde (0.96 g, 10 mmol) was slowly added dropwise into the solution. The reaction mixture was then heated to 35 ℃ and kept for 2 h, yielding a yellow solid. After collection and multiple washes with distilled water, the solid was dissolved into DCM. Then the solution was washed with saturated NaCl aqueous solution and saturated NaHSO_3_ aqueous solution. The organic layer was purified by column chromatography, affording a yellow solution. Removal of the solvent under reduced pressure yielded the title compound S1 as a yellow solid (1.7 g, 77%).

***5-(furan-2-ylmethylene)-2,2-dimethyl-1,3-dioxane-4,6-dione (S2)***


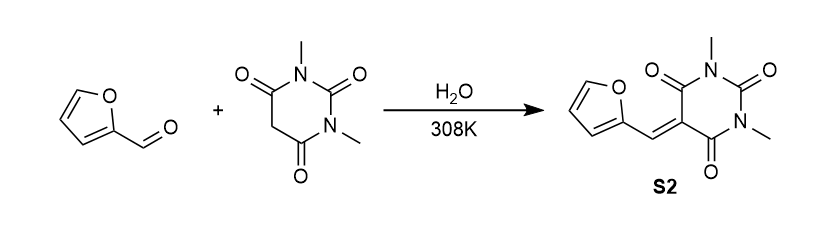


1,3-Dimethylpyrimidine-2,4,6(1H,3H,5H)-trione (1.56 g, 10 mmol) was dissolved in distilled water (50 mL) with stirring. Furfural (0.96 g, 10 mmol) was added dropwise to the solution. The reaction mixture was heated to 35 °C and stirred for 2 hours, yielding a yellow solid. The solid was collected by filtration, washed with distilled water, and dissolved in dichloromethane (DCM). The resulting organic solution was then washed sequentially with saturated aqueous NaCl and saturated aqueous NaHSO₃. The organic layer was purified by column chromatography. After concentration of the eluent under reduced pressure, compound **S2** was obtained as a yellow solid (2.0 g, 86% yield).

***DASA-1 (D1)***

Compound **S1** (2.22 g, 10 mmol) was dissolved in dichloromethane (DCM, 50 mL). To this solution, N-propylaniline (1.35 g, 10 mmol) was added dropwise. The reaction mixture was stirred at 40 °C for 2 hours under light-protected conditions. The solution color changed from pale yellow to a deep bluish-purple during the reaction, indicating the formation of the product. The reaction progress was monitored by thin-layer chromatography (TLC). Upon completion, the mixture was purified by silica gel column chromatography to afford **D1** as a deep purple solid (2.39 g, 67% yield).

***DASA-2 (D2)***


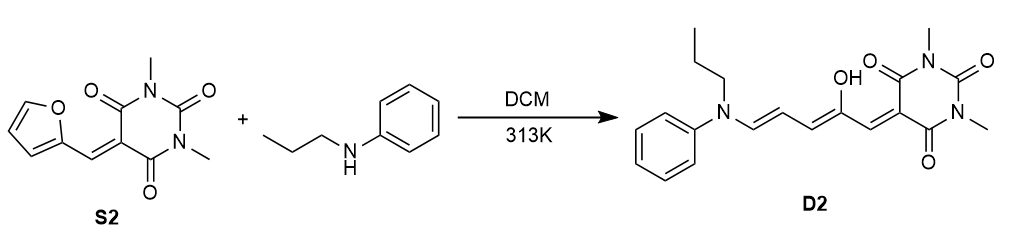


Compound **S2** (2.32 g, 10 mmol) was dissolved into 50 mL of DCM and slowly added *N-*propylaniline (1.56 g, 10 mmol), the reaction turned to deep purple after stirring at 40 °C for 2 h. Then the reaction solution was condensed by rotary evaporation to obtain the crude product and purified by column chromatography to obtain the red solid as pure product **D2** (2.14 g, 58% yield).

**^1^H NMR** (400 MHz, CDCl_3_) δ (ppm): δ 12.40 (s, 1H), 7.68 (t, *J* = 8.0 Hz, 1H), 7.45 (t, *J* = 7.3 Hz, 2H), 7.34 (s, 1H), 7.23 (d, *J* = 8.7 Hz, 2H), 6.77 (d, *J* = 3.9 Hz, 1H), 6.69 (d, *J* = 3.9 Hz, 1H), 6.43 (t, *J* = 7.2 Hz, 1H), 3.82 (t, *J* = 8 Hz, 2H), 3.35 (s, 3H), 1.75 (m, 2H) , 0.98 (t, *J* = 7.4 Hz, 6H), 0.93 (*cyclic*).

**^13^C NMR** (101 MHz, CDCl_3_) δ (ppm): 206.21, 167.17, 166.75, 163.54, 151.25, 150.76, 148.46, 134.28, 130.06, 129.34, 120.53, 116.77, 63.09, 49.91, 46.96, 46.44, 28.77, 19.87, 12.19.

**HRMS:** *m/z* found 368.9742 ([**M-H**]⁺); calcd for C_20_H_23_N_3_O_4_ 369.1689.

***DASA-3 (D3)***

Compound **S1** (2.22 g, 10 mmol) was dissolved in dichloromethane (DCM, 50 mL). To this solution, indoline (1.19 g, 10 mmol) was added dropwise, resulting in an immediate color change to a deep blue. The reaction mixture was then stirred at 40 °C for 2 hours. After completion, the solvent was removed under reduced pressure, and the crude product was purified by column chromatography to afford **D3** as a deep blue solid (2.62 g, 77% yield).

# Photophysical properties of DASAs and fluorescent polymers

## Photoisomerization of DASAs

**Table S1** Isomerization kinetics of **D1**-**D3** upon visible light irradiation (Above the dotted line) and heat treatment (RT, below the dotted line) in DCM.

| Time | D1 | D2 | D3 |
| --- | --- | --- | --- |
| (s) |  |  |  |
| 0 | 0.00 | 0.00 | 0.00 |
| 4 | 42.15 | 38.89 | 42.68 |
| 8 | 68.19 | 70.75 | 57.72 |
| 12 | 80.10 | 81.90 | 66.32 |
| 16 | 85.57 | 87.53 | 79.27 |
| 20 | 94.45 | 90.85 | 86.79 |
| 40 | 98.74 | 97.08 | 95.03 |
| 100 | 99.26 | 99.89 | 99.12 |
| 200 | 99.26 | 99.79 | 99.28 |
| 300 | 98.96 | 99.49 | 99.02 |
| 400 | 98.96 | 99.49 | 99.02 |
| 500 | 94.45 | 94.67 | 98.72 |
| 700 | 88.60 | 87.13 | 98.36 |
| 1000 | 77.81 | 74.67 | 97.59 |
| 2000 | 64.42 | 59.79 | 96.67 |
| 3000 | 37.27 | 31.55 | 94.31 |


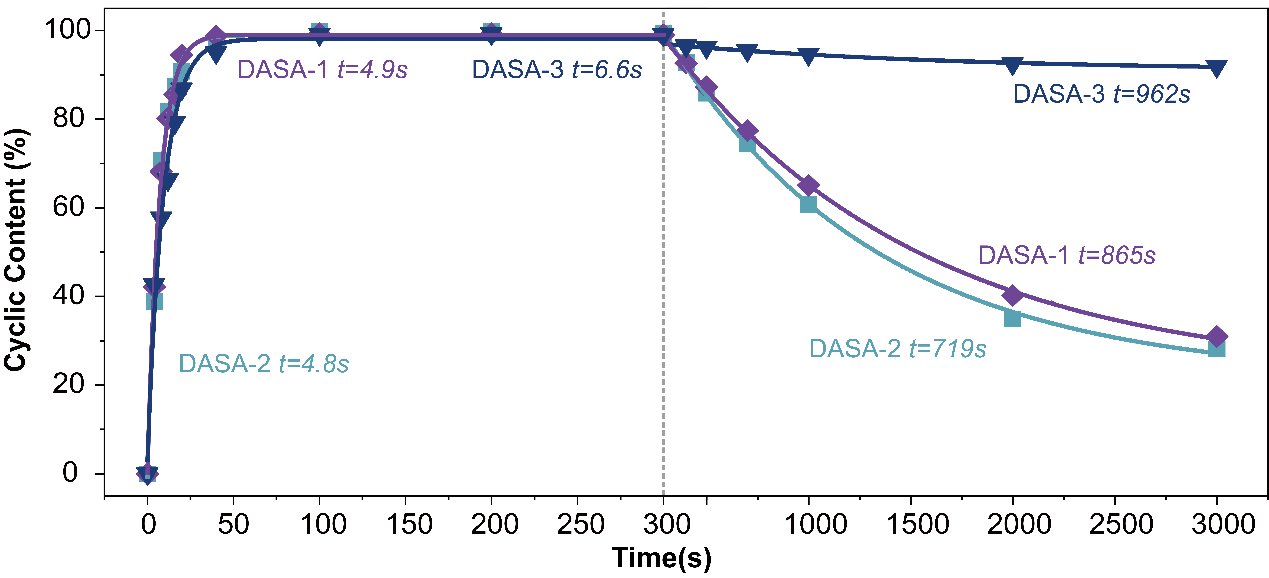


**Figure S1** First-order *linear*-to-*cyclic* kinetics of **D1-D3** under visible light irradiation (0-300 s, 20 mW/cm^2^) and subsequent *cyclic*-to-*linear* thermal relaxation (300-3000 s, 20 °C).

**Table S2** Fitting results of isomerization kinetic of **D1-D3** upon visible light irradiation and heat treatment in DCM.

|  | Visible light irradiation | | Heat treatment | |
| --- | --- | --- | --- | --- |
|  | t | R^2^ | t | R^2^ |
|  | (s) |  | (s) |  |
| D1 | 4.9 | 0.99863 | 865 | 0.99913 |
| D2 | 4.8 | 0.99591 | 719 | 0.99835 |
| D3 | 6.6 | 0.99035 | 962 | 0.99595 |

## Fluorescence re-absorption by DASAs


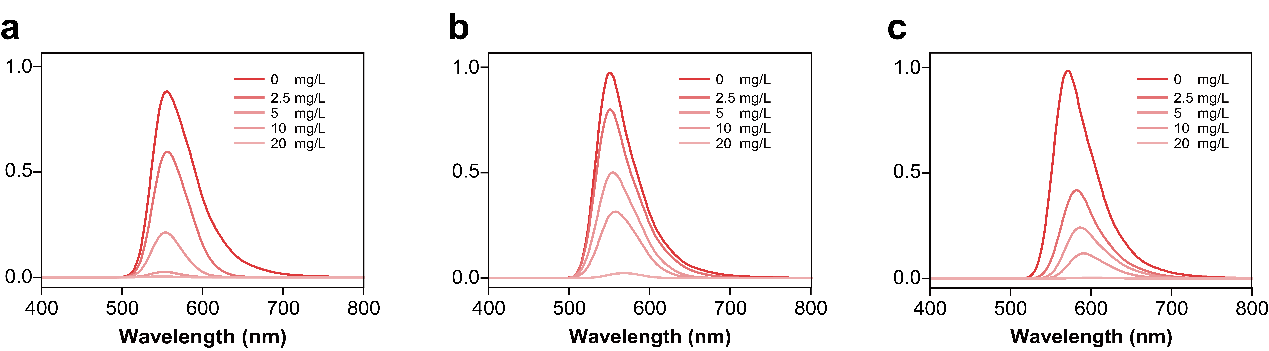


**Figure S2** Normalized fluorescence spectra of **PFO** (2.5 mg/L in DCM)after sequentially adding a) **D1**, b) **D2**, and c) **D3**, the concentration of DASAs is increased from 0 to 1000 mg/L.


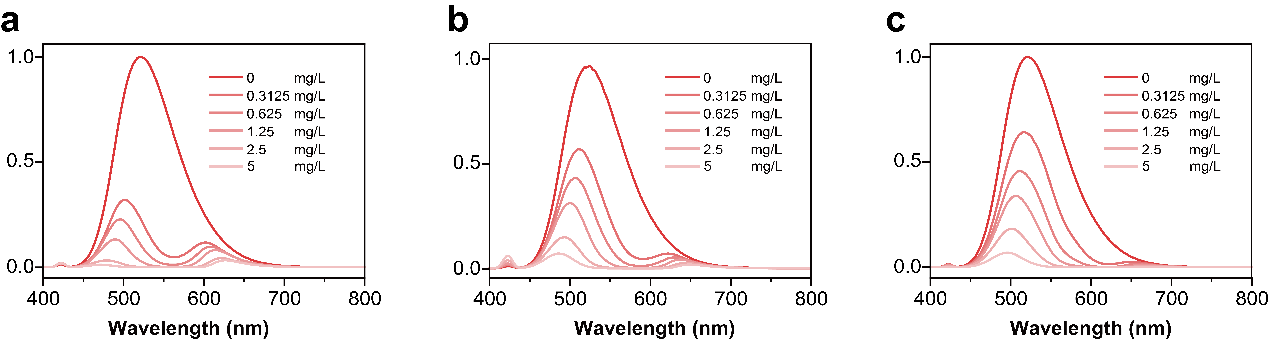


**Figure S3** Normalized fluorescence spectra of **F8BT** (2.5 mg/L in DCM) after sequentially adding a) **D1**, b) **D2**, and c) **D3**, the concentration of DASAs is increased from 0 to 1000 mg/L.


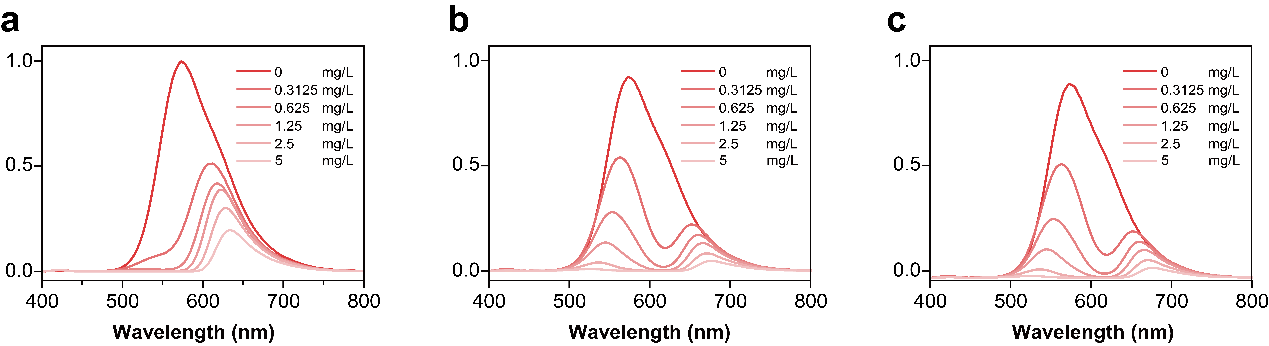


**Figure S4** Normalized fluorescence spectra of **P3HT** (2.5 mg/L in DCM) after sequentially adding a) **D1**, b) **D2**, and c) **D3**, the concentration of DASAs is increased from 0 to 1000 mg/L.


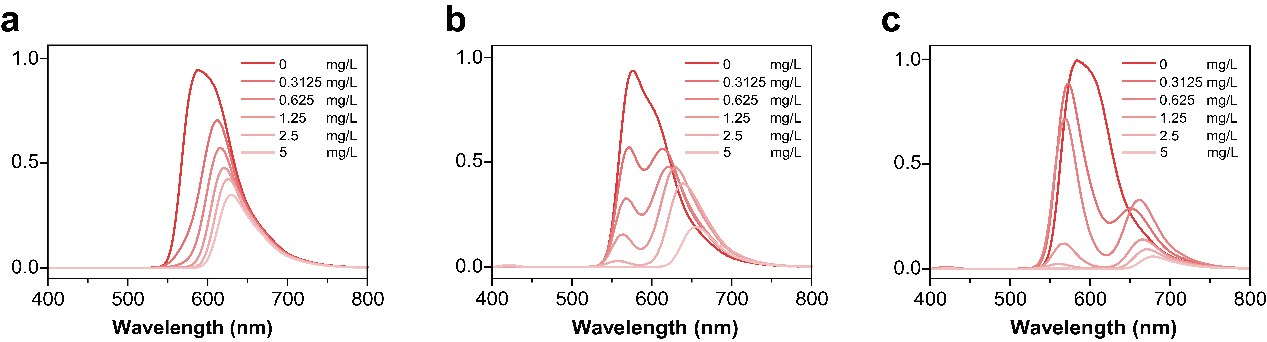


**Figure S5** Normalized fluorescence spectra of **MEH-PPV** (2.5 mg/L in DCM) after sequentially adding a) **D1**, b) **D2**, and c) **D3**, the concentration of DASAs is increased from 0 to 1000 mg/L.

Gradual addition of DASAs into the solutions of fluorescent polymers induces attenuation of the photoluminescence, which follows the one-phase exponential decay function:

$$\begin{aligned} PL={PL}_{e}+A\timesⅇ^{\left( -\frac{c}{c_{e}} \right)}\#(1) \end{aligned}$$

where $PL$ and ${PL}_{e}$ represent the relative fluorescence intensity at a DASAs concentration $c$ and at equilibrium; $c_{e}$ denotes the DASAs concentration required to reach the equilibrium; the concentration to obtain 50% attenuation (c_1/2_) is calculated through $c_{1/2}=ln2\times c_{e}$.

**Table S3** Summary of fitted information for the quenching plots of polymer fluorescence by DASAs.

| DASAs | Polymers | c_1/2_  (mg/mL) | PL_e_  (%) | R^2^ |
| --- | --- | --- | --- | --- |
| D1 | PFO | 4.327 | 0 | 0.9891 |
|  | F8BT | 0.071 | 1.1 | 0.9976 |
|  | MEH-PPV | 0.133 | 0.5 | 0.9998 |
|  | P3HT | 0.135 | 0 | 0.9999 |
| D2 | PFO | 1.068 | 26.4 | 0.9977 |
|  | F8BT | 0.355 | 3.76 | 0.9833 |
|  | MEH-PPV | 0.392 | 0.2 | 0.9994 |
|  | P3HT | 0.161 | 0 | 0.9996 |
| D3 | PFO | 0.403 | 0 | 0.9102 |
|  | F8BT | 0.428 | 6.9 | 0.9803 |
|  | MEH-PPV | 0.712 | 0 | 0.9344 |
|  | P3HT | 0.311 | 0 | 0.9971 |

## Overlapping between the absorption and emission spectra

**
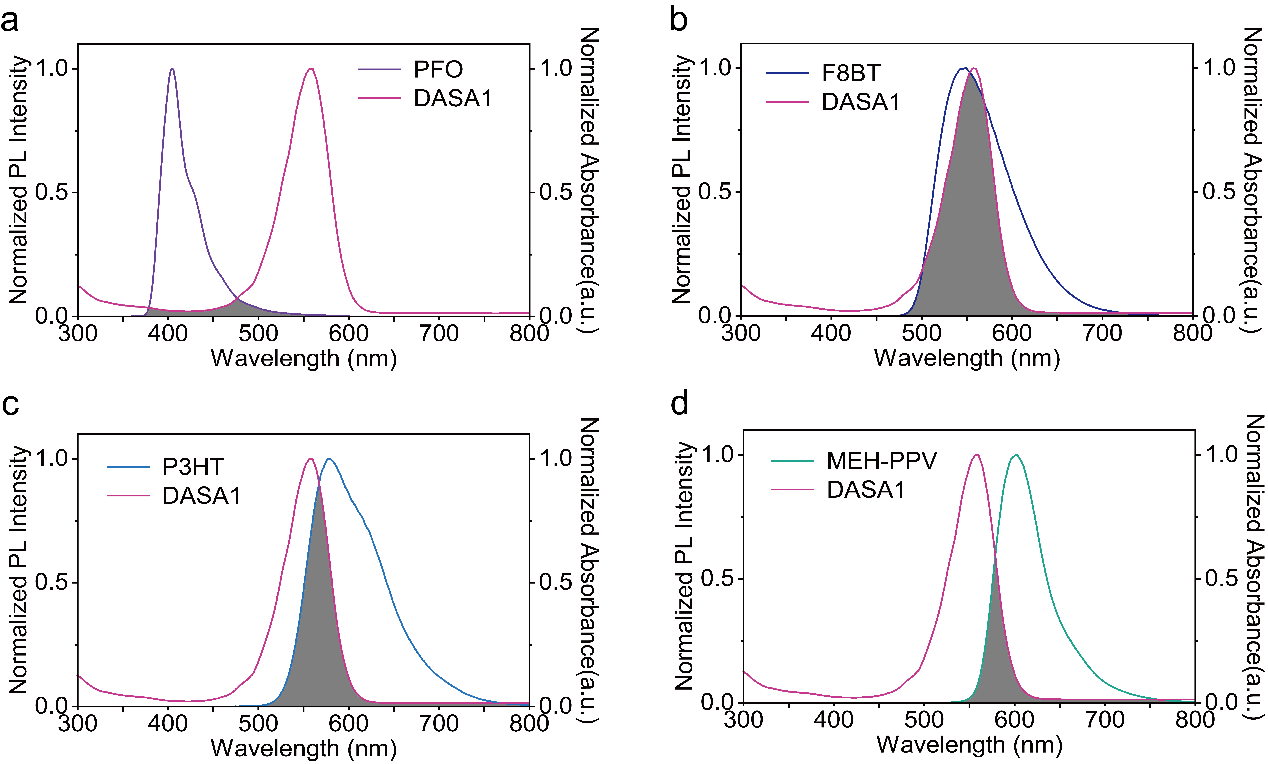
**

**Figure S6** Overlapping between the normalized absorption spectrum of **D1** and the normalized emission spectra of a) **PFO**, b) **F8BT**, c) **P3HT**, and d) **MEH-PPV**.

**
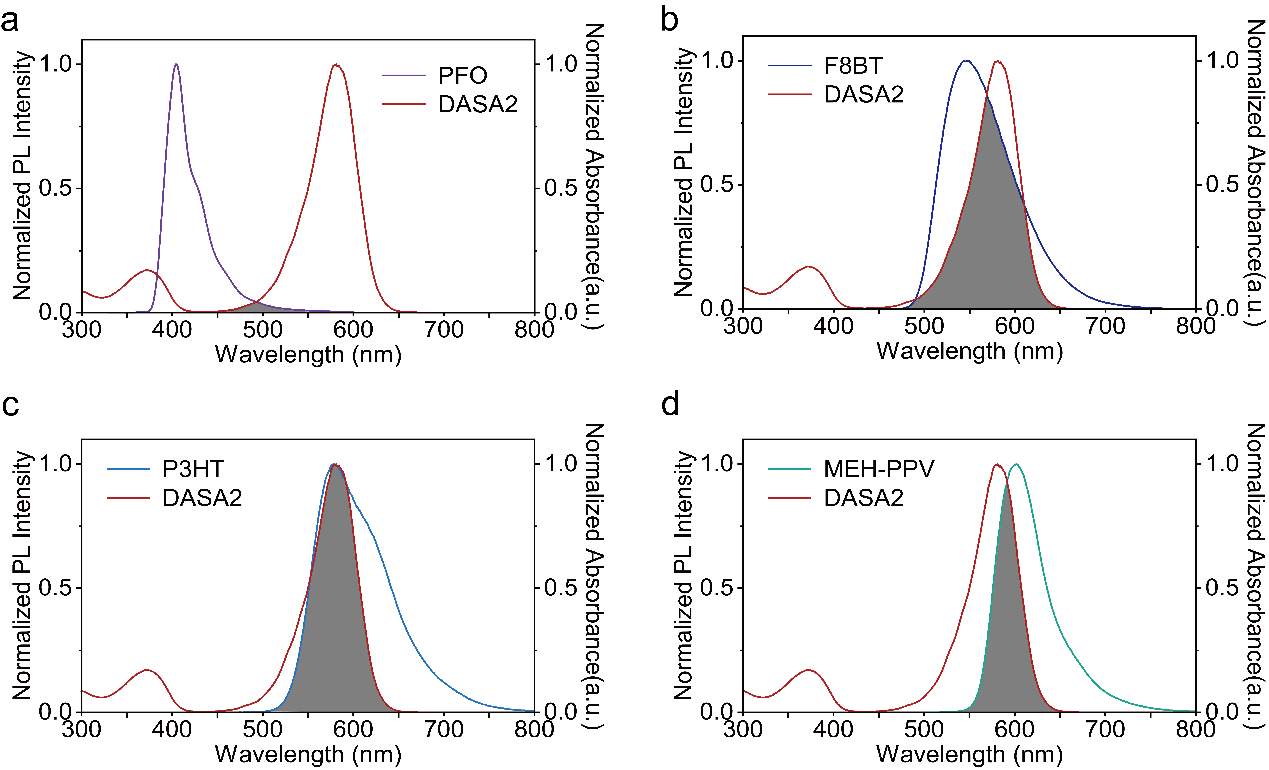
**

**Figure S7** Overlapping between the normalized absorption spectrum of **D2** and the normalized emission spectra of a) **PFO**, b) **F8BT**, c) **P3HT**, and d) **MEH-PPV**.

**
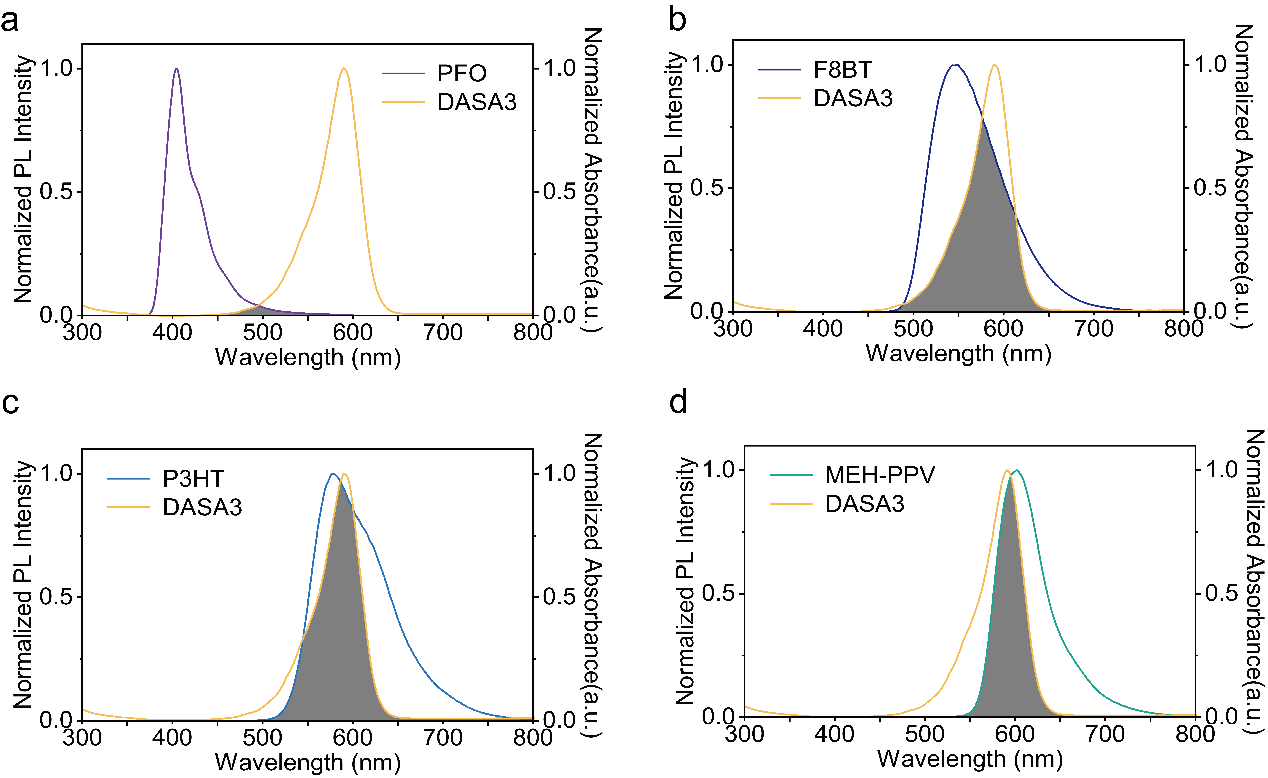
**

**Figure S8** Overlapping between the normalized absorption spectrum of **D3** and the normalized emission spectra of a) **PFO**, b) **F8BT**, c) **P3HT**, and d) **MEH-PPV**.

**Table S4** Summary of $A_{i}$, $A_{u}$ and spectral overlap parameter (R%) obtained from Figure S6-S8.

| DASAs | polymers | A_i_ | A_u_ | R% |
| --- | --- | --- | --- | --- |
| D1 | PFO | 5.14 | 106.50 | 0.048 |
|  | F8BT | 60.24 | 99.80 | 0.604 |
|  | MEH-PPV | 16.00 | 121.10 | 0.132 |
|  | P3HT | 36.49 | 127.30 | 0.287 |
| D2 | PFO | 4.40 | 106.30 | 0.041 |
|  | F8BT | 54.96 | 105.60 | 0.520 |
|  | MEH-PPV | 34.20 | 101.57 | 0.337 |
|  | P3HT | 58.45 | 100.30 | 0.583 |
| D3 | PFO | 1.39 | 100.40 | 0.014 |
|  | F8BT | 46.40 | 103.20 | 0.450 |
|  | MEH-PPV | 35.40 | 91.20 | 0.388 |
|  | P3HT | 49.80 | 102.58 | 0.485 |

# Photodynamic properties in solutions

## Dynamics of *linear*-to-*cyclic* isomerization of DASAs

**
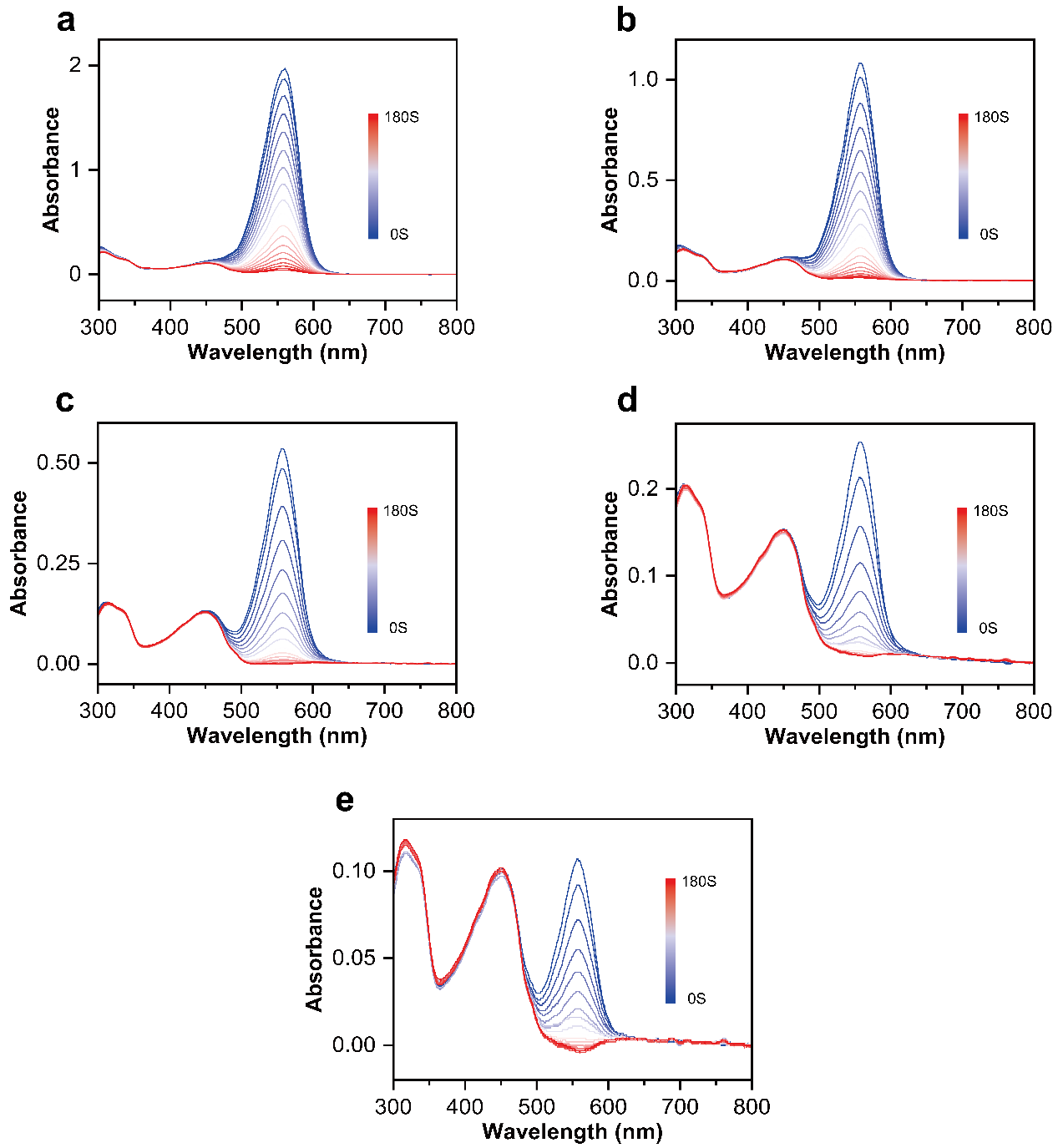
**

**Figure S9** Time-dependent UV/vis absorption spectra of **D1/F8BT** upon 420 nm light irradiation. The concentration of **F8BT** was kept 2.5 mg/L in DCM, while **D1** was dissolved with different concentrations: a) 20 mg/L, b) 10 mg/L, c) 5 mg/L, d) 2.5 mg/L, and e) 1.25 mg/L.

**
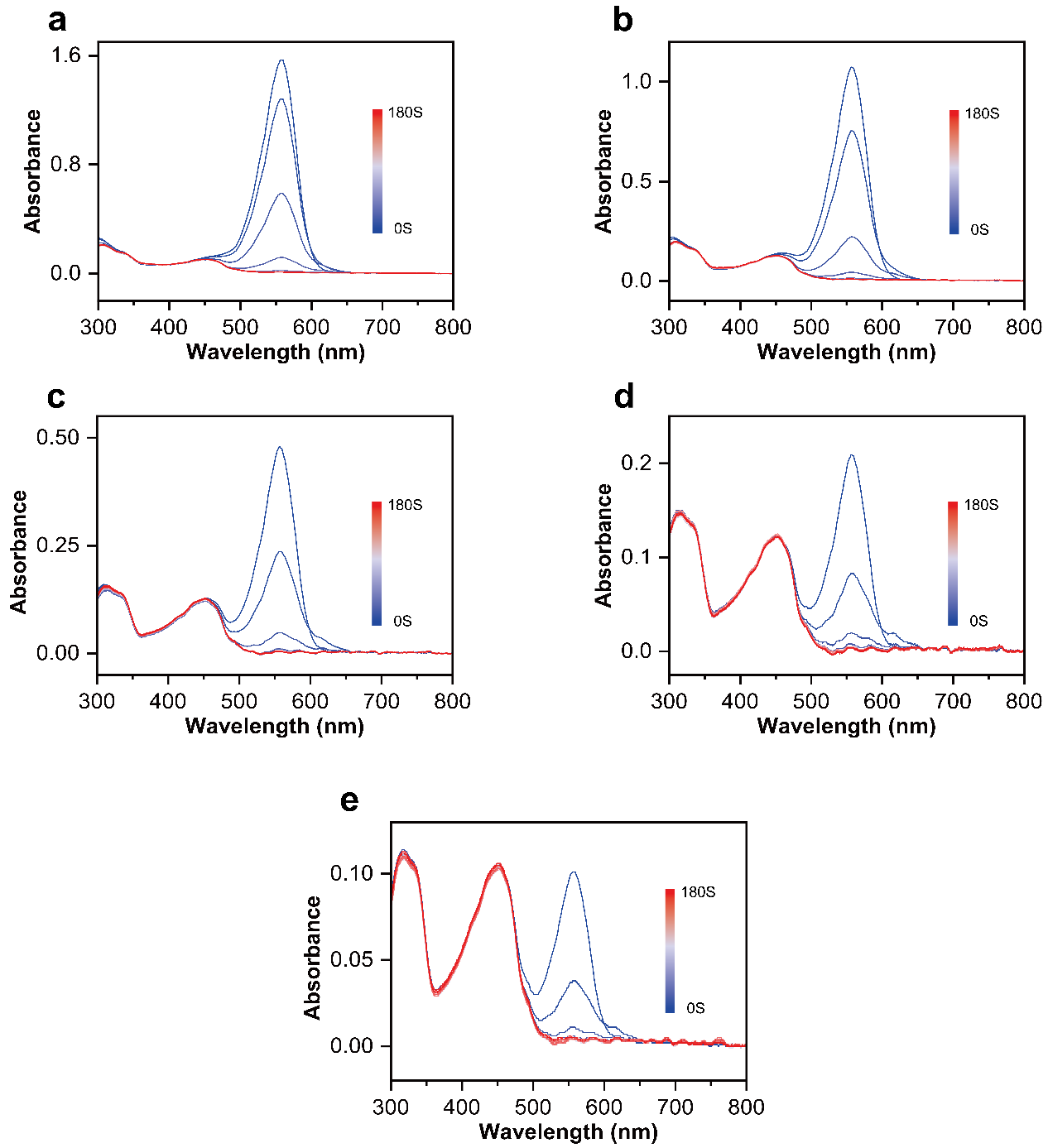
**

**Figure S10** Time-dependent UV/vis absorption spectra of **D1/F8BT** upon 520 nm light irradiation. The concentration of **F8BT** was kept 2.5 mg/L in DCM, while **D1** was dissolved with different concentrations: a) 20 mg/L, b) 10 mg/L, c) 5 mg/L, d) 2.5 mg/L, and e) 1.25 mg/L.

**
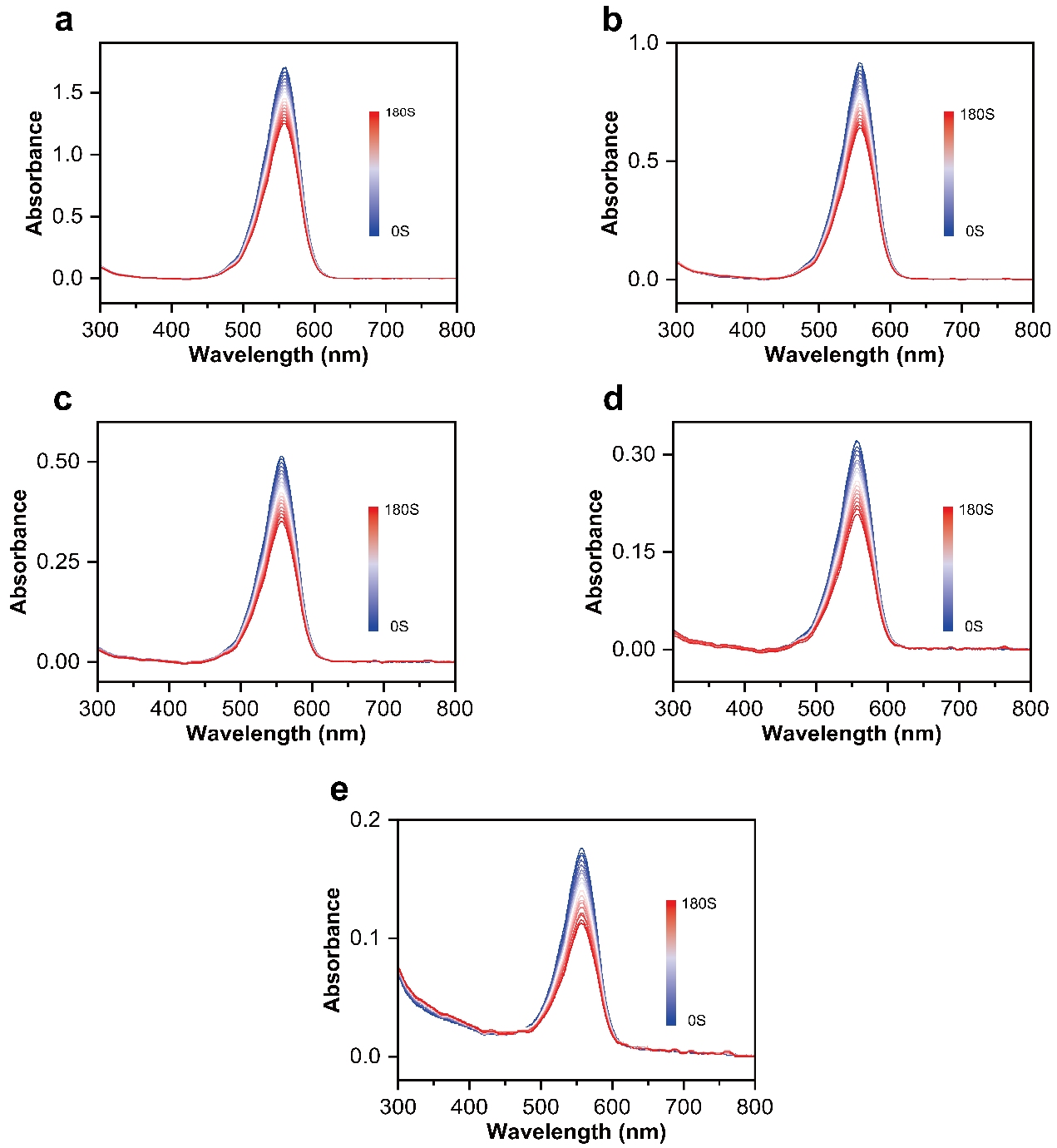
**

**Figure S11** Time-dependent UV/vis absorption spectra of **D1** upon 420 nm light irradiation (20 mW/cm^2^). **D1** was dissolved in DCM with different concentrations: a) 20 mg/L, b) 10 mg/L, c) 5 mg/L, d) 2.5 mg/L, and e) 1.25 mg/L.


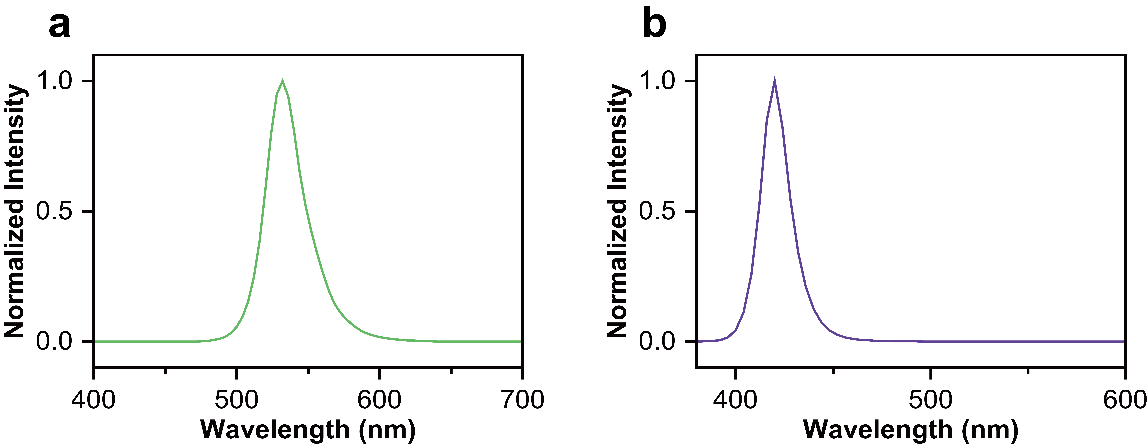


**Figure S12** Emission spectra of a) 520 nm green light-emitting diode (LED) and b) 420 nm violet LED.

The *linear*-to-*cyclic* isomerization of **D1/F8BT** under 420 nm and 520 nm light irradiation follows the first-order kinetics, which could be quantitatively investigated through the following equation:

$$\begin{aligned} L=L_{e}+A\timesⅇ^{\left( -\frac{t}{t_{0}} \right)}\#(2) \end{aligned}$$

where $L$ and $L_{e}$ represent the *linear* **D1** content at an irradiation time $t$ and at equilibrium, respectively; $t_{0}$ denotes the irradiation time required to reach the equilibrium; the rate constant (k) is calculated through $k=1/t_{0}$.

**Table S5** Summary for the fitted information for the *linear*-to-*cyclic* isomerization of **D1/F8BT**.

| D1 concentration  (mg/L) | Condition | k  (s^-1^) | L_e_  (%) | R^2^ |
| --- | --- | --- | --- | --- |
| 1.25 | 420 nm w/o F8BT | 0.0006 | - | 0.9973 |
|  | 420 nm w/ F8BT | 0.026 | 0 | 0.9987 |
|  | 520 nm w/ F8BT | 0.181 | 0.6 | 0.9986 |
| 2.5 | 420 nm w/o F8BT | 0.0004 | - | 0.9995 |
|  | 420 nm w/ F8BT | 0.023 | 0 | 0.9991 |
|  | 520 nm w/ F8BT | 0.161 | 0.3 | 0.9998 |
| 5 | 420 nm w/o F8BT | 0.0003 | - | 0.9997 |
|  | 420 nm w/ F8BT | 0.018 | - | 0.9978 |
|  | 520 nm w/ F8BT | 0.132 | 0 | 0.9965 |
| 10 | 420 nm w/o F8BT | 0.0002 | - | 0.9998 |
|  | 420 nm w/ F8BT | 0.014 | - | 0.9974 |
|  | 520 nm w/ F8BT | 0.088 | 0 | 0.9879 |
| 20 | 420 nm w/o F8BT | 0.0002 | - | 0.9967 |
|  | 420 nm w/ F8BT | 0.007 | - | 0.9828 |
|  | 520 nm w/ F8BT | 0.067 | 0 | 0.9999 |

## *in-situ* emission spectra of D1/F8BT solutions

**
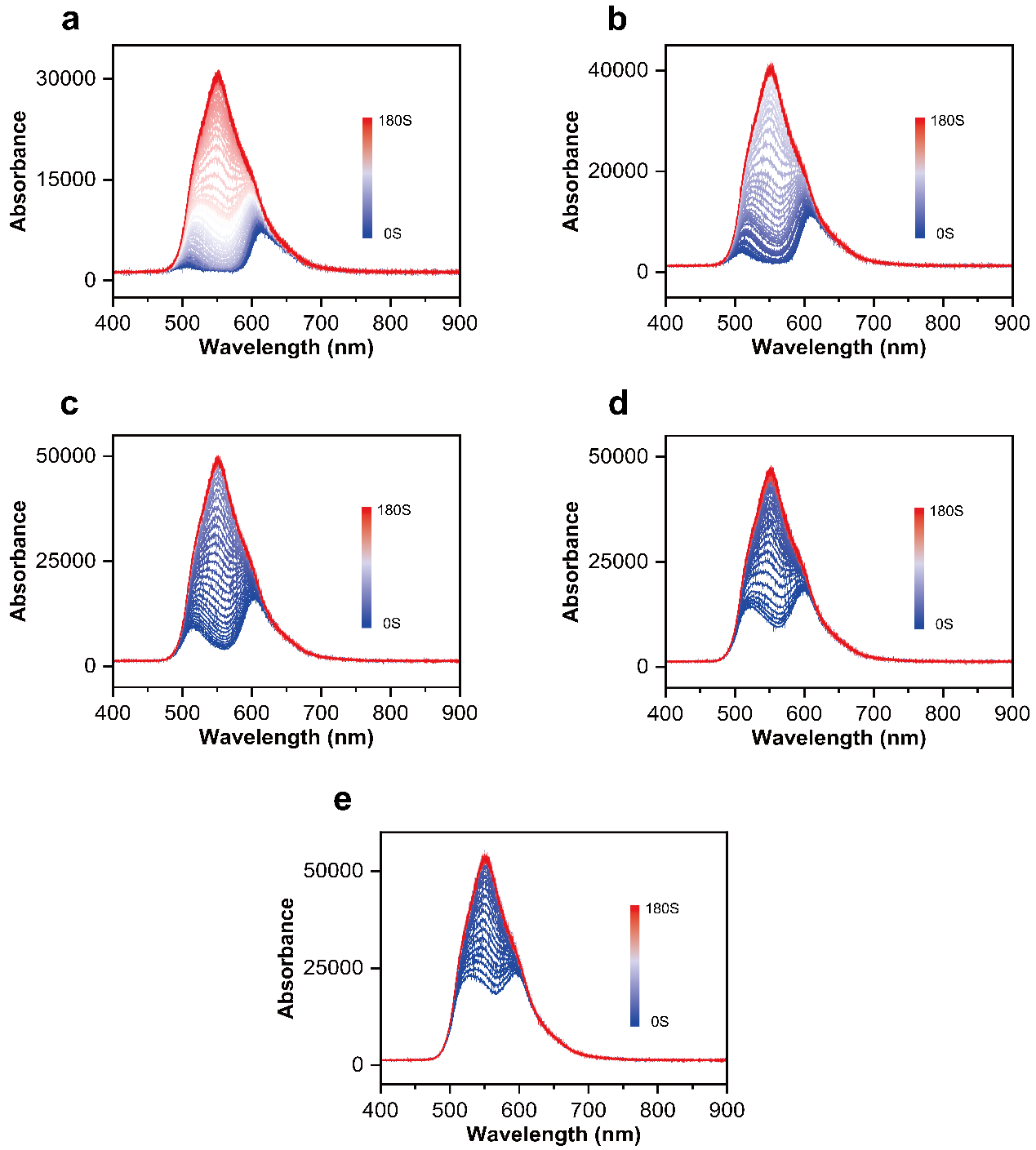
**

**Figure S13** Time-dependent *in-situ* emission spectra of **DASAs/F8BT** under 420 nm violet light irradiation (20 mW/cm^2^). The concentration of **F8BT** was kept 2.5 mg/L in DCM, while **D1** was dissolved with different concentrations: a) 20 mg/L, b) 10 mg/L, c) 5 mg/L, d) 2.5 mg/L, and e) 1.25 mg/L. Excitation: 365 nm.

**
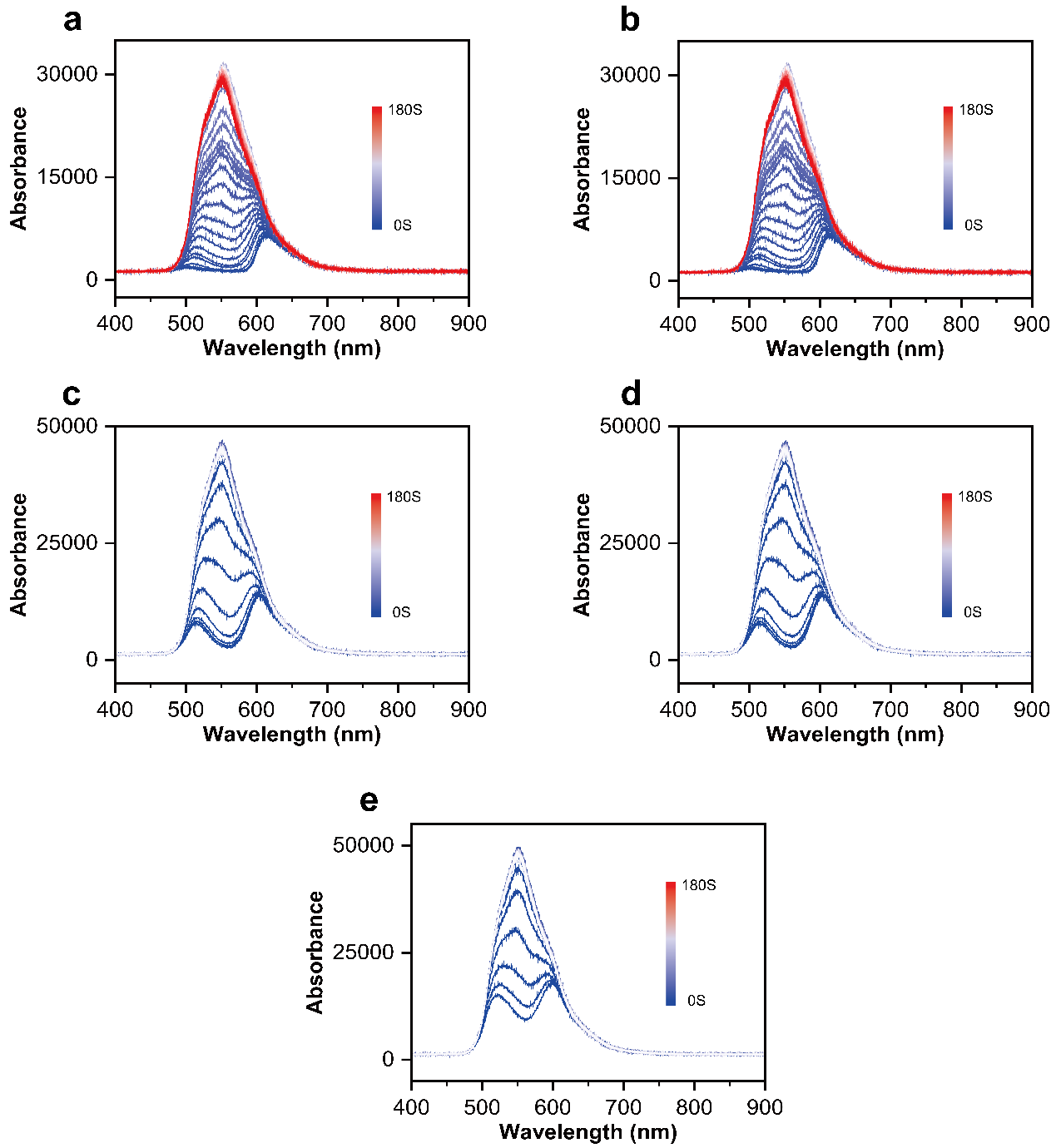
**

**Figure S14** Time-dependent *in-situ* emission spectra of **DASAs/F8BT** under 520 nm green light irradiation (20 mW/cm^2^). The concentration of **F8BT** was kept 2.5 mg/L in DCM, while **D1** was dissolved with different concentrations: a) 20 mg/L, b) 10 mg/L, c) 5 mg/L, d) 2.5 mg/L, and e) 1.25 mg/L. Excitation: 365 nm.

The “turning on” process of fluorescence under 420 nm and 520 nm light irradiation follows the similar first-order kinetics as described in Eq. 2.

**Table S6** Summary of fitted information for the “turning on” process of fluorescence.

| D1 concentration  (mg/L) | Irradiation  (nm) | k  (s^-1^) | R^2^ |
| --- | --- | --- | --- |
| 1.25 | 420 | 0.064 | 0.9759 |
|  | 520 | 0.421 | 0.9966 |
| 2.5 | 420 | 0.050 | 0.9761 |
|  | 520 | 0.195 | 0.9960 |
| 5 | 420 | unfitted | unfitted |
|  | 520 | unfitted | unfitted |
| 10 | 420 | unfitted | unfitted |
|  | 520 | unfitted | unfitted |
| 20 | 420 | unfitted | unfitted |
|  | 520 | unfitted | unfitted |

# Photoisomerization dynamic properties in solid

## Deposition of ester-functionalized compounds on surface

**
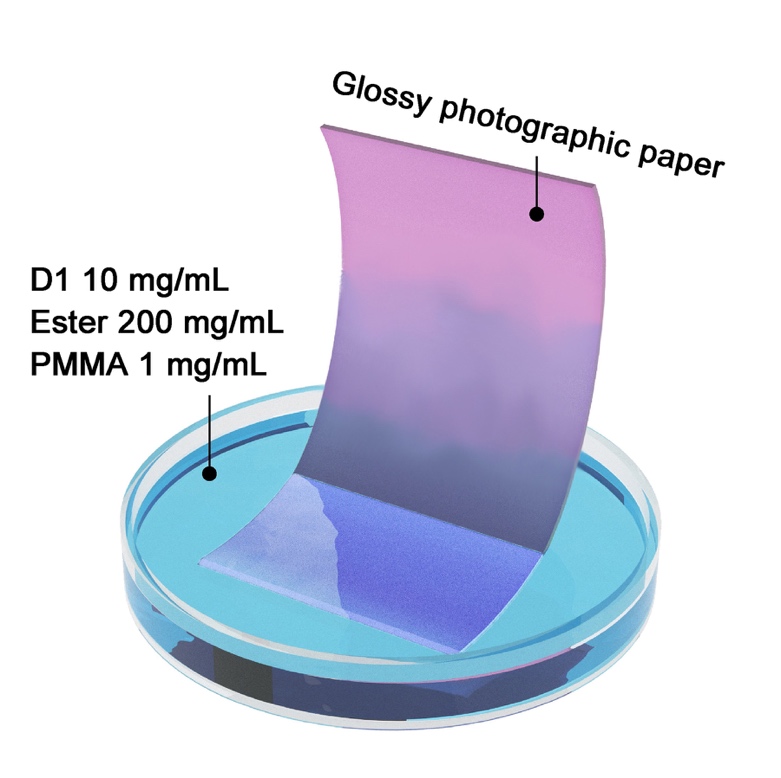
**

**Figure S15** Schematic illustration of co-depositing **D1** (10 mg/mL), ester-functionalized compounds (200 mg/mL) and polymethyl methacrylate (PMMA, 1 mg/mL) onto glossy photographic paper surface.

To investigate the solid-state photochromism of DASAs under assistance of ester-functionalized compounds, D1 was co-deposited with various ester-functionalized compounds onto glossy photographic paper via solution soaking.

1. **D1**, ester-functionalized compounds, and PMMA were dissolved into DCM with the concentration of 10, 200, and 1 mg/mL, respectively.
2. A glossy photographic paper was soaked into the resulted solution and kept for 30 s, after which the paper was dried under room temperature for 5 min until the evaporation of DCM.

Interestingly, because of the intermolecular aggregation, **D1** might show slight color shifts on paper surface after solvent drying.

## Photochromism dynamics promoted by ester-functionalized compounds

**
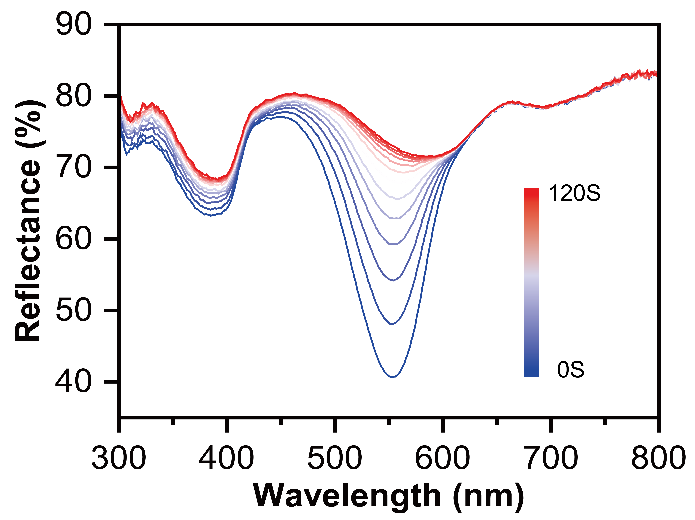
**

**Figure S16** Diffuse-reflectance spectra of photographic paper treated by **D1** (10 mg/mL in DCM, with 200 mg/mL **DM**) under 520 nm light irradiation (20 mW/cm^2^).

**
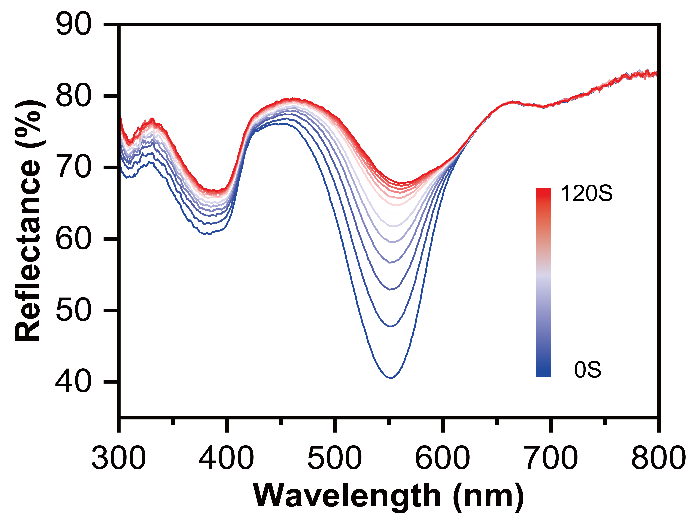
**

**Figure S17** Diffuse-reflectance spectra of photographic paper treated by **D1** (10 mg/mL in DCM, with 200 mg/mL **EAA**) under 520 nm light irradiation (20 mW/cm^2^).

**
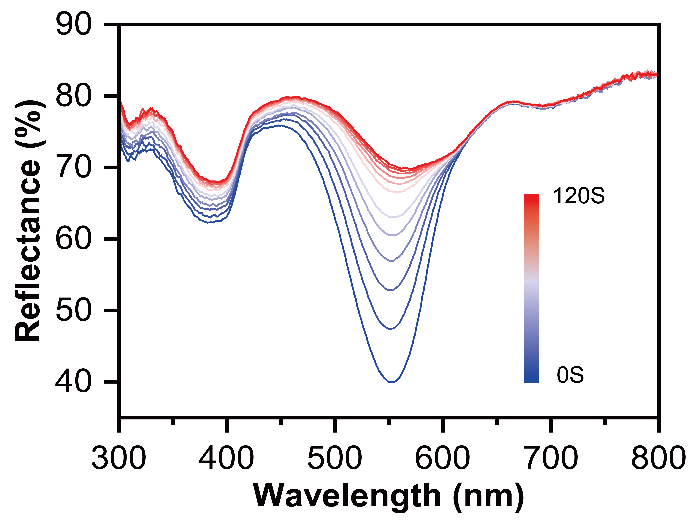
**

**Figure S18** Diffuse-reflectance spectra of photographic paper treated by **D1** (10 mg/mL in DCM, with 200 mg/mL **HB**) under 520 nm light irradiation (20 mW/cm^2^).

**
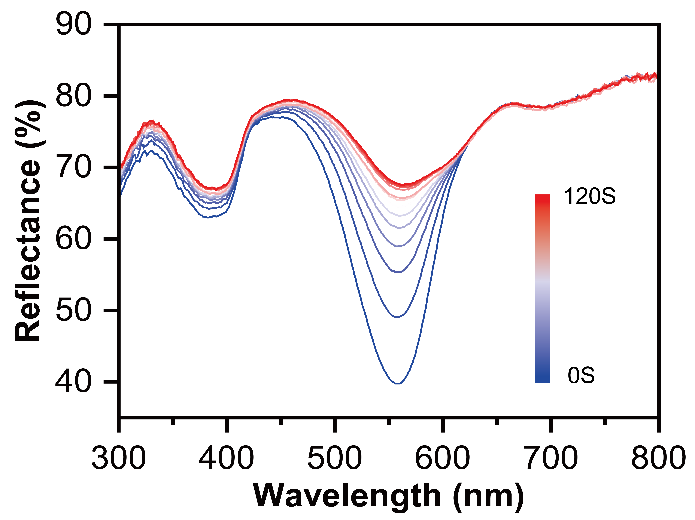
**

**Figure S19** Diffuse-reflectance spectra of photographic paper treated by **D1** (10 mg/mL in DCM, with 200 mg/mL **PB**) under 520 nm light irradiation (20 mW/cm^2^).

The solid-state photochromism of **D1** under 520 nm light irradiation follows the first-order kinetics, which is similar to the *linear*-to-*cyclic* isomerization of **D1** and could be quantitatively investigated through the following equation:

$$\begin{aligned} R=R_{e}+A\timesⅇ^{\left( -\frac{t}{t_{0}} \right)}\#(3) \end{aligned}$$

where $R$ and $R_{e}$ represent the reflectance values at an irradiation time $t$ and at equilibrium, respectively; $t_{0}$ denotes the irradiation time required to reach the equilibrium; the rate constant (k) is calculated through $k=1/t_{0}$.

**
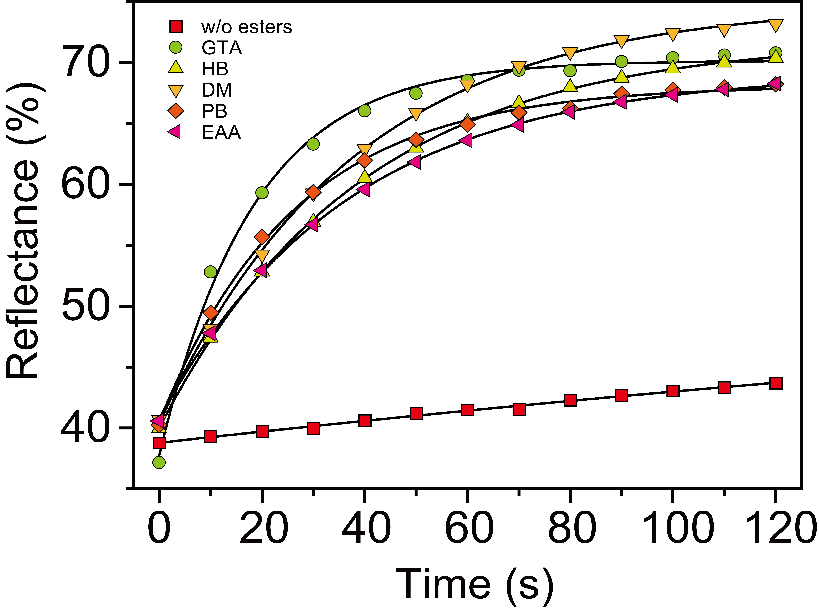
**

**Figure S20** First-order kinetics of solid-state photochromism of **D1** under 520 nm light irradiation (20 mW/cm^2^). Ester-functionalized compounds (200 mg/mL for each) were added.

**Table S7** Summary of fitted information for the photochromism on surface under assistance of ester-functionalized compounds.

| Esters | k  (s^-1^) | R_e_  (%) | R^2^ |
| --- | --- | --- | --- |
| PB | 0.038 | 64.8 | 0.9985 |
| DM | 0.026 | 74.3 | 0.9995 |
| GTA | 0.055 | 70.2 | 0.9961 |
| EAA | 0.027 | 66.3 | 0.9998 |
| HB | 0.025 | 71.9 | 0.9999 |
| w/o | 0.002 | 43.3 | 0.9941 |

## dynamics promoted by GTA with various concentrations


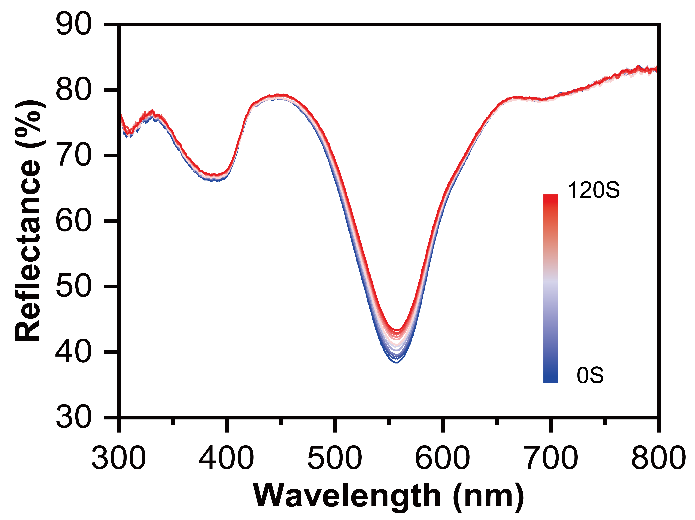


**Figure S21** Diffuse-reflectance spectra of photographic paper treated by **D1** (10 mg/mL in DCM, with 0 mg/mL **GTA**) under 520 nm light irradiation (20 mW/cm^2^).


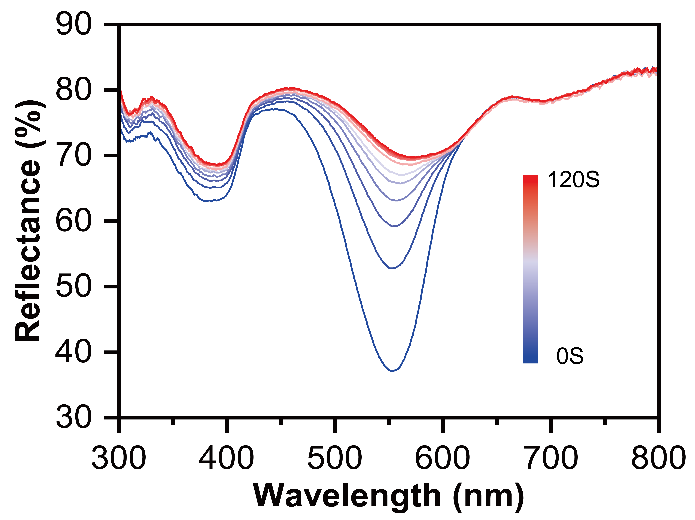


**Figure S22** Diffuse-reflectance spectra of photographic paper treated by **D1** (10 mg/mL in DCM, with 200 mg/mL **GTA**) under 520 nm light irradiation (20 mW/cm^2^).


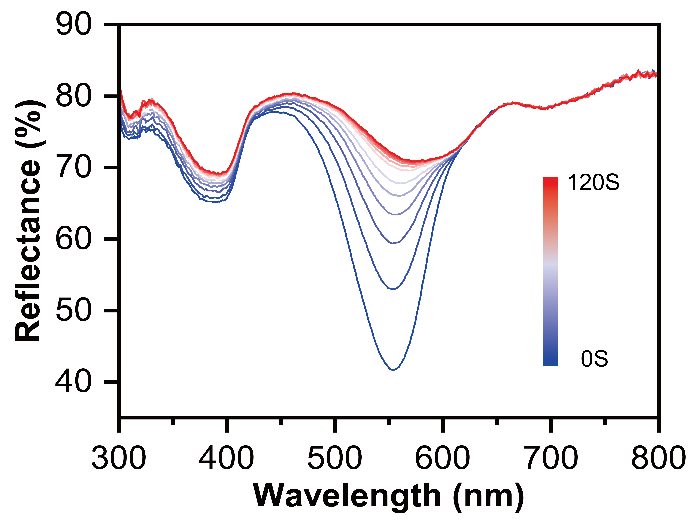


**Figure S23** Diffuse-reflectance spectra of photographic paper treated by **D1** (10 mg/mL in DCM, with 100 mg/mL **GTA**) under 520 nm light irradiation (20 mW/cm^2^).


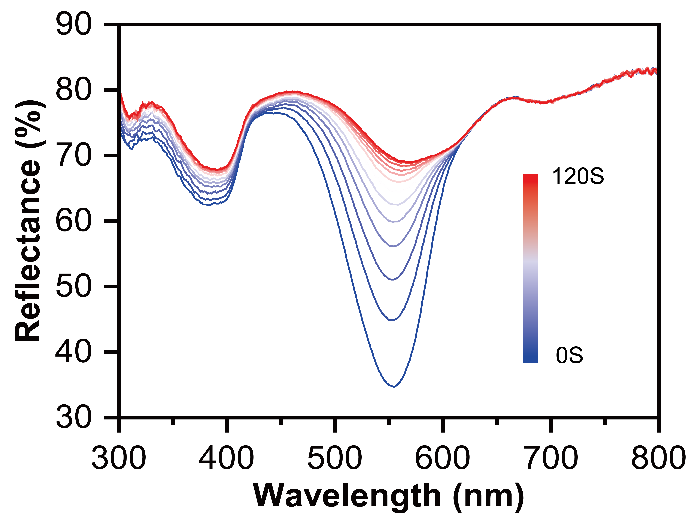


**Figure S24** Diffuse-reflectance spectra of photographic paper treated by **D1** (10 mg/mL in DCM, with 50 mg/mL **GTA**) under 520 nm light irradiation (20 mW/cm^2^).


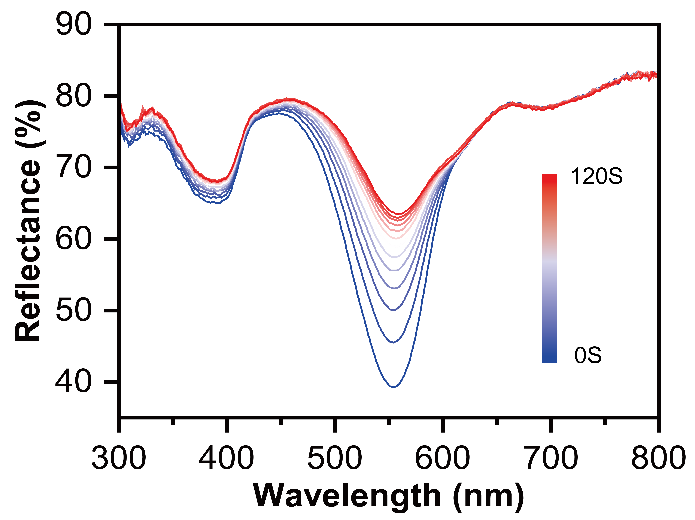


**Figure S25** Diffuse-reflectance spectra of photographic paper treated by **D1** (10 mg/mL in DCM, with 25 mg/mL **GTA**) under 520 nm light irradiation (20 mW/cm^2^).


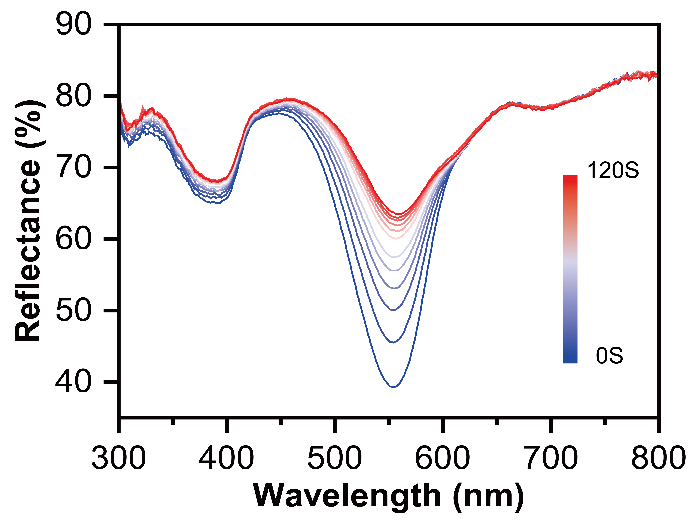


**Figure S26** Diffuse-reflectance spectra of photographic paper treated by **D1** (10 mg/mL in DCM, with 12.5 mg/mL **GTA**) under 520 nm light irradiation (20 mW/cm^2^).





**Figure S27** First-order kinetics of solid-state photochromism of **D1** under 520 nm light irradiation (20 mW/cm^2^). **GTA** with various concentrations were added.

**Table S8** Summary of fitted information for the photochromism on surface under assistance of **GTA** with various concentrations.

| GTA concentrations  (mg/mL) | k  (s^-1^) | R_e_  (%) | R^2^ |
| --- | --- | --- | --- |
| 0 | 0.002 | 43.3 | 0.9941 |
| 12.5 | 0.024 | 63.9 | 0.9963 |
| 25 | 0.025 | 64.7 | 0.9991 |
| 50 | 0.029 | 70.7 | 0.9997 |
| 100 | 0.043 | 71.9 | 0.9991 |
| 200 | 0.055 | 70.2 | 0.9961 |


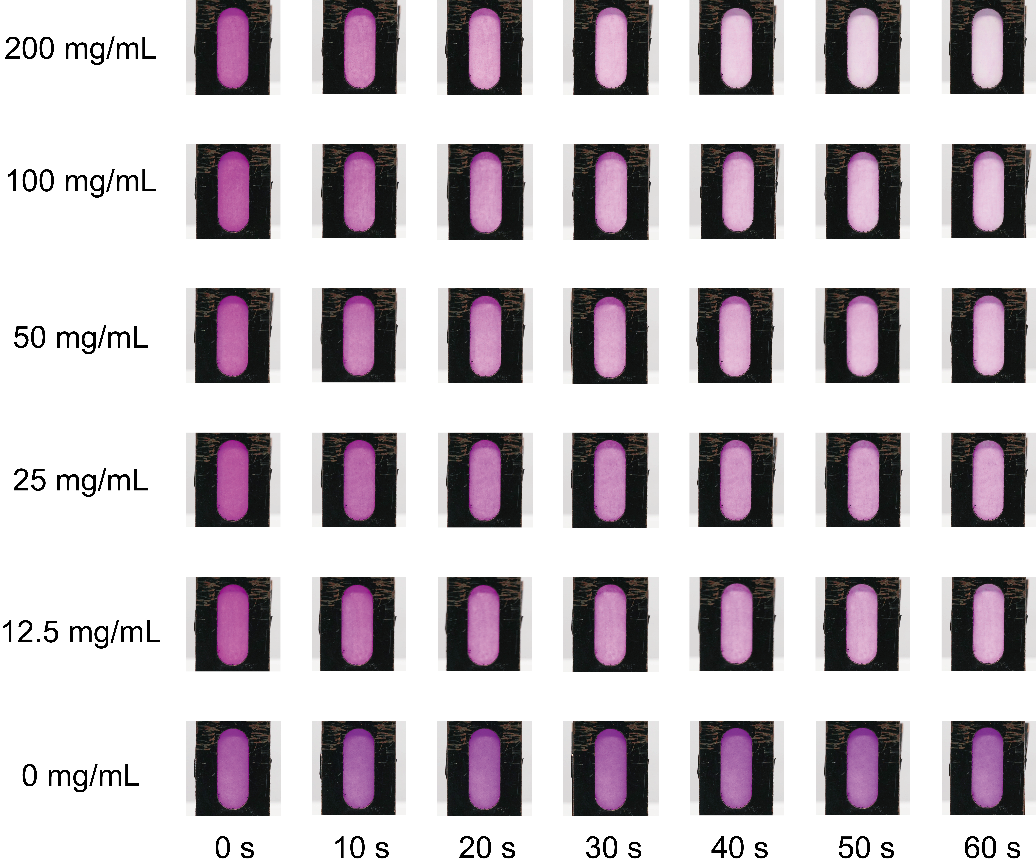


**Figure S28** Photographic images on surface pretreated by **GTA** with various concentrations under 520 nm light irradiation (20 mW/cm^2^). **GTA** concentrations: 200, 100, 50, 25, 12.5, and 0 mg/mL. Irradiation time: 0, 10, 20, 30, 40, 50, and 60 s.

## Photoisomerization dynamics controlled by grayscale


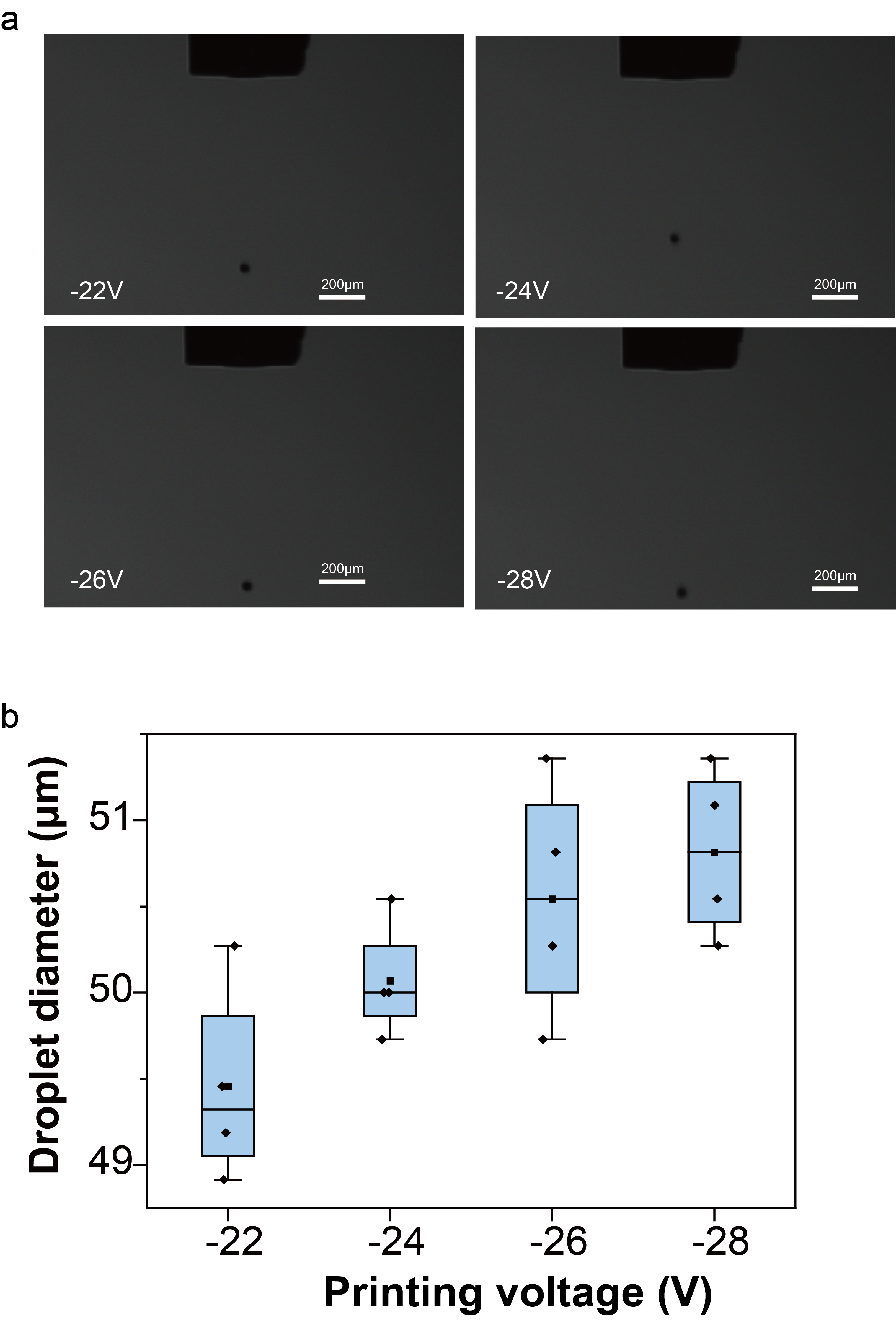


**Figure S29** a) Photographic images of droplets printed from a piezoelectric nozzle, the printing voltage is controlled between -22 and -28 V; b) Distribution of the droplet’s diameter under various printing voltages.


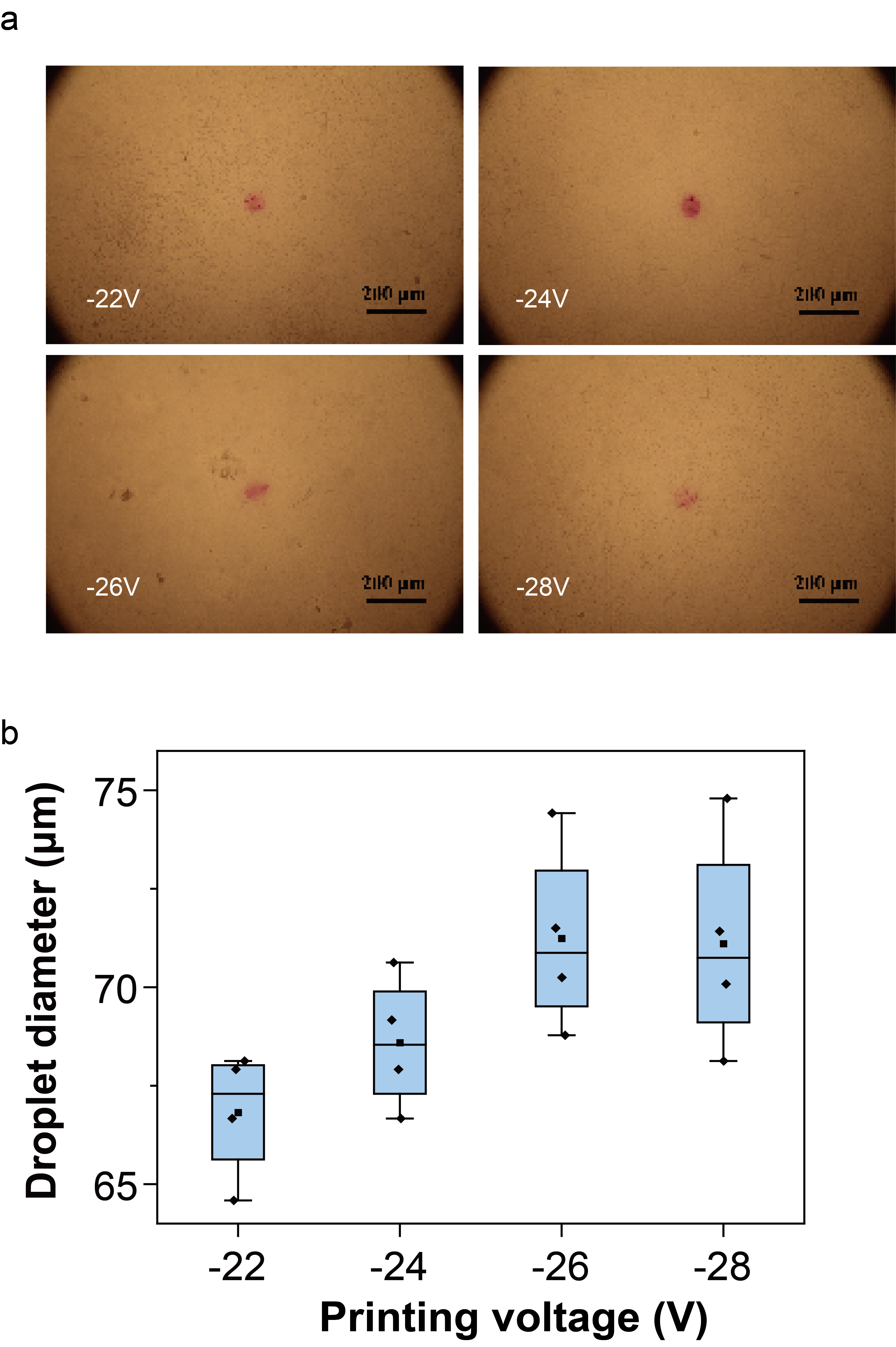


**Figure S30** a) Optical microscopic images of printed pixels on glossy photographic paper surface, the printing voltage is controlled between -22 and -28 V; b) Distribution of the pixel’s diameter under various printing voltages.


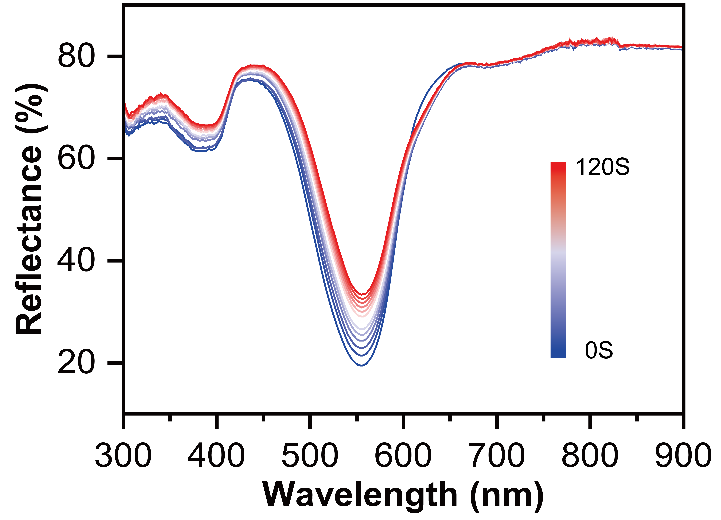


**Figure S31** Diffuse-reflectance spectra of photographic paper treated by **D1** (10 mg/mL in DCM) under 520 nm light irradiation (20 mW/cm^2^). The ester-functionalized ink (**GTA** 200 mg/mL in ethanol) was coated with the grayscale value of 0%.


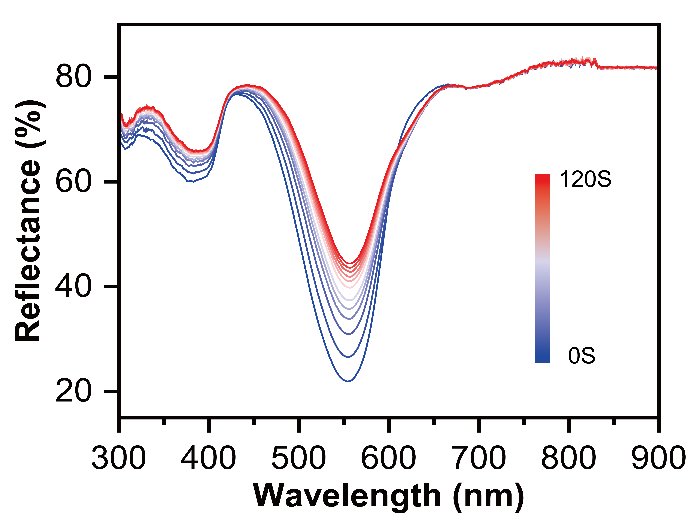


**Figure S32** Diffuse-reflectance spectra of photographic paper treated by **D1** (10 mg/mL in DCM) under 520 nm light irradiation (20 mW/cm^2^). The ester-functionalized ink (**GTA** 200 mg/mL in ethanol) was printed with the grayscale value of 20%.


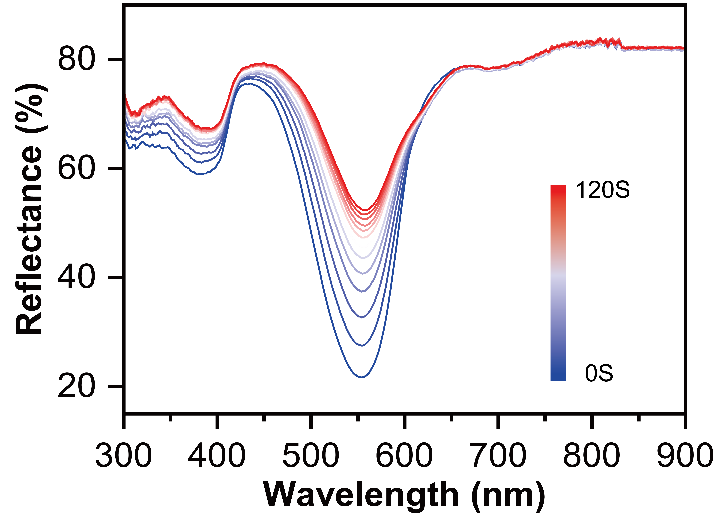


**Figure S33** Diffuse-reflectance spectra of photographic paper treated by **D1** (10 mg/mL in DCM) under 520 nm light irradiation (20 mW/cm^2^). The ester-functionalized ink (**GTA** 200 mg/mL in ethanol) was printed with the grayscale value of 40%.


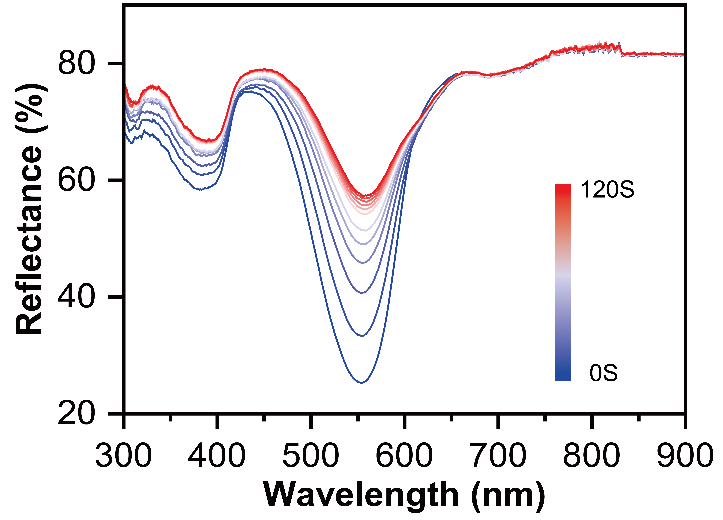


**Figure S34** Diffuse-reflectance spectra of photographic paper treated by **D1** (10 mg/mL in DCM) under 520 nm light irradiation (20 mW/cm^2^). The ester-functionalized ink (**GTA** 200 mg/mL in ethanol) was printed with the grayscale value of 80%.


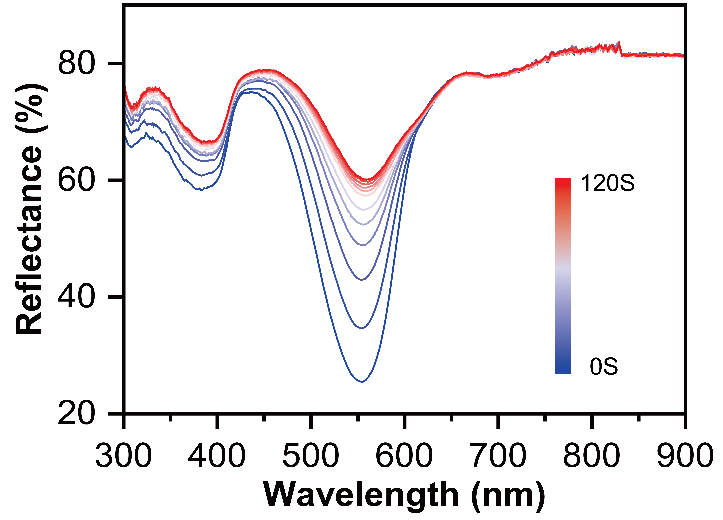


**Figure S35** Diffuse-reflectance spectra of photographic paper treated by **D1** (10 mg/mL in DCM) under 520 nm light irradiation (20 mW/cm^2^). The ester-functionalized ink (**GTA** 200 mg/mL in ethanol) was coated with the grayscale value of 100%.

**Table S9** Summary of fitted information for the photochromism on surface with various grayscale values.

| Grayscale values  (%) | k  (s^-1^) | R_e_  (%) | R^2^ |
| --- | --- | --- | --- |
| 0 | 0.002 | 43.3 | 0.9941 |
| 20 | 0.020 | 50.6 | 0.9981 |
| 40 | 0.021 | 56.8 | 0.9995 |
| 80 | 0.033 | 66.9 | 0.9997 |
| 100 | 0.041 | 72.9 | 0.9977 |

Because the photochromic and non-photochromic dots in the microarray is below the resolution of human eyes, the photochromism on surface arises from the integrated response of the photochromic and non-photochromic dots. The reflectance value in a specific area relies on the average of the photochromic and non-photochromic pixels, which is closely interrelated with the grayscale values.

**
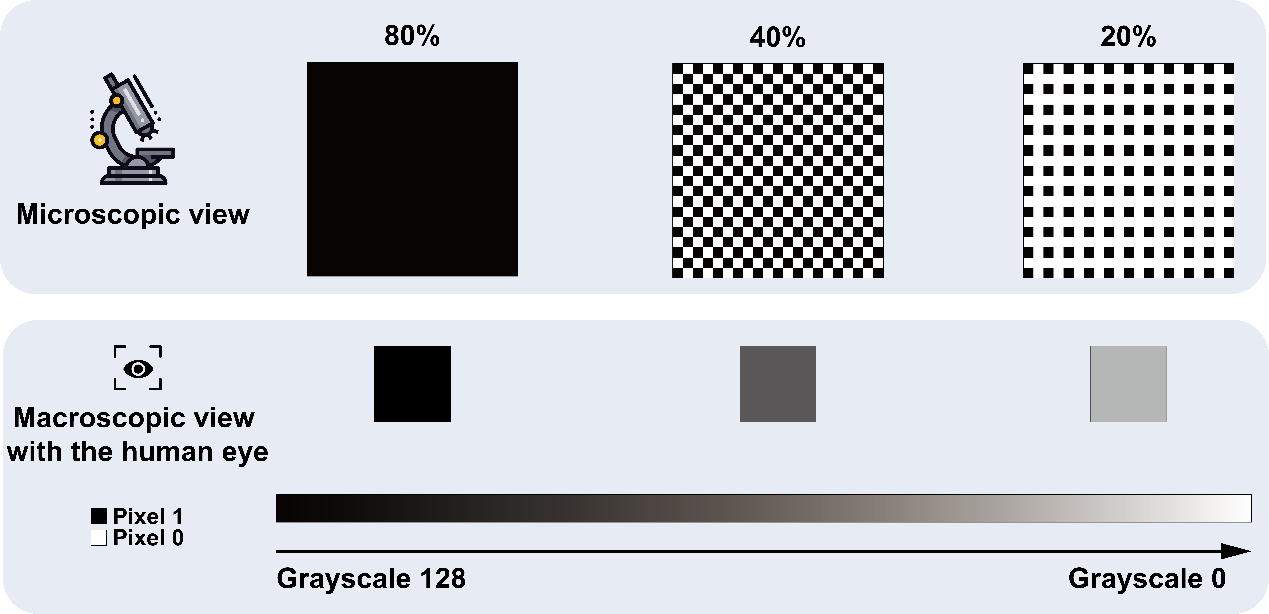
**

**Figure S36** Schematic illustration of the integrated effect of photochromic and non-photochromic dots.


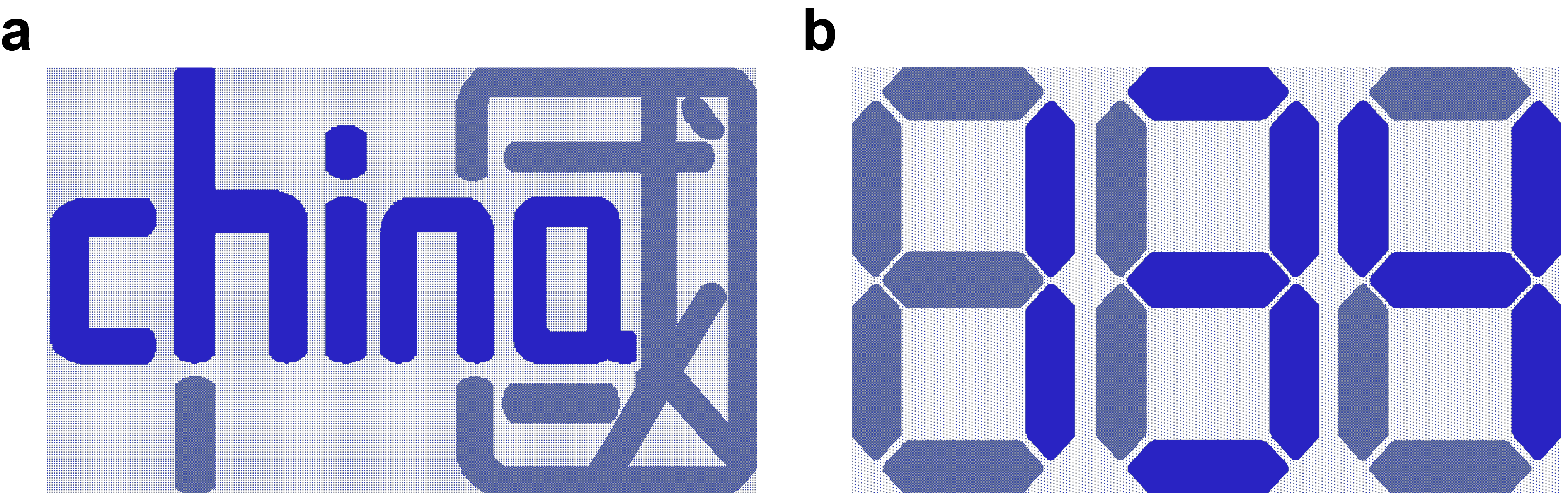


**Figure S37** Schematic illustration of grayscale patterns of a) "china" and b) "134" printed on photographic paper.


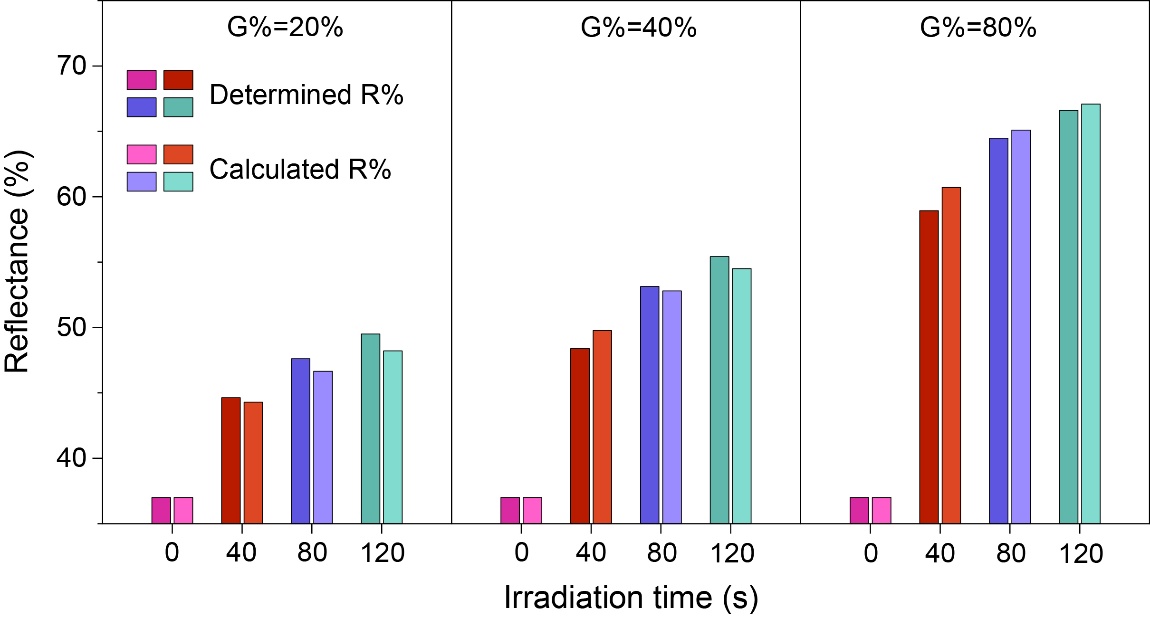


**Figure S38** Calculated and determined R_G_ on surface under 520 nm light irradiation (20 mW/cm^2^). Grayscale values: 20%, 40% and 80%; Irradiation time: 0 s, 40 s, 80 s and 120 s.

**
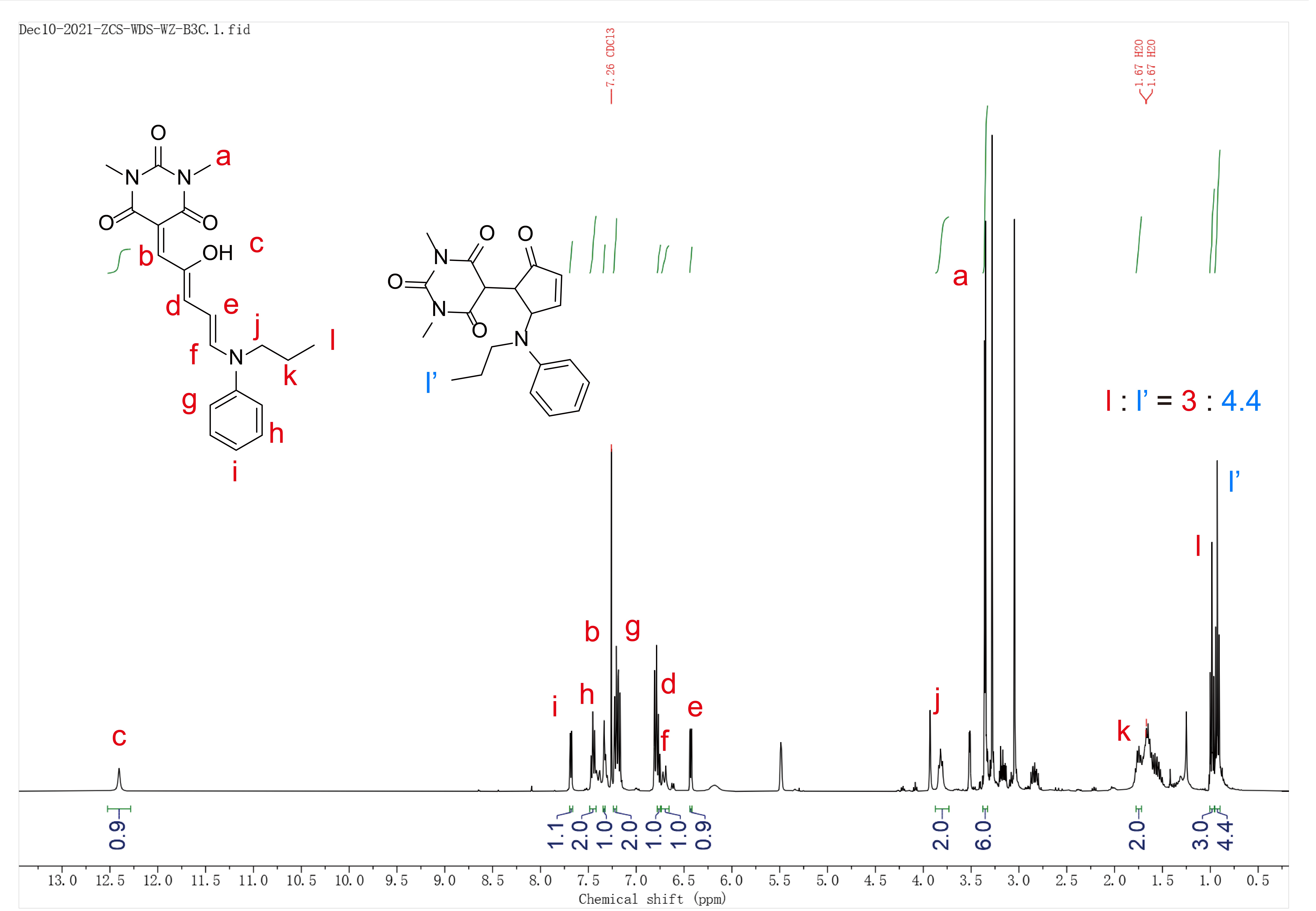
**

**Figure S39** ^1^H NMR spectrum of DASA-2 (400 MHz, 298 K), [DASA-2] = 10 mM, solvent: CDCl_3_.

**
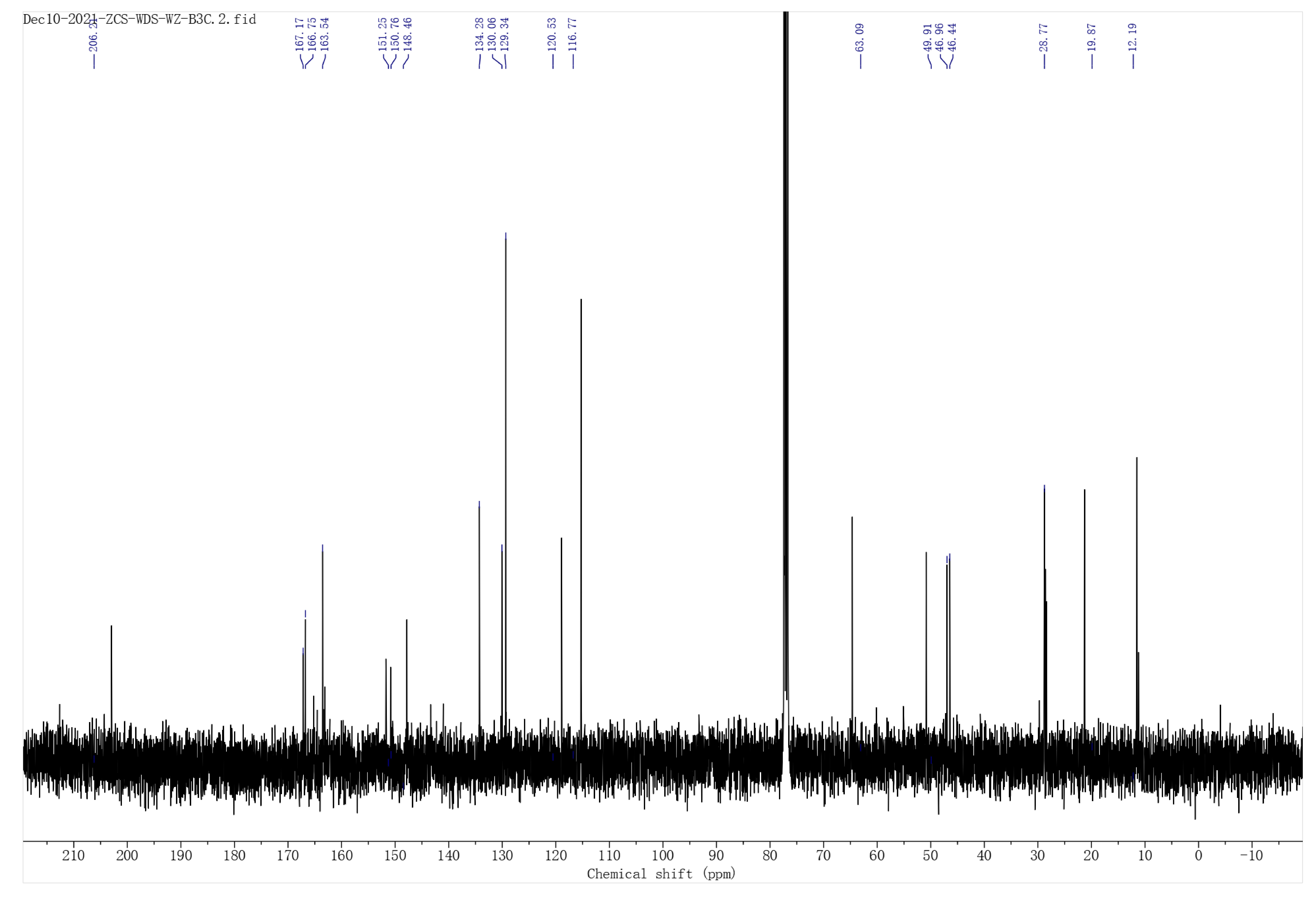
**

**Figure S40** ^13^C NMR spectrum of DASA-2 (101 MHz, 298 K), [DASA-2] = 10 mM, solvent: CDCl_3_.


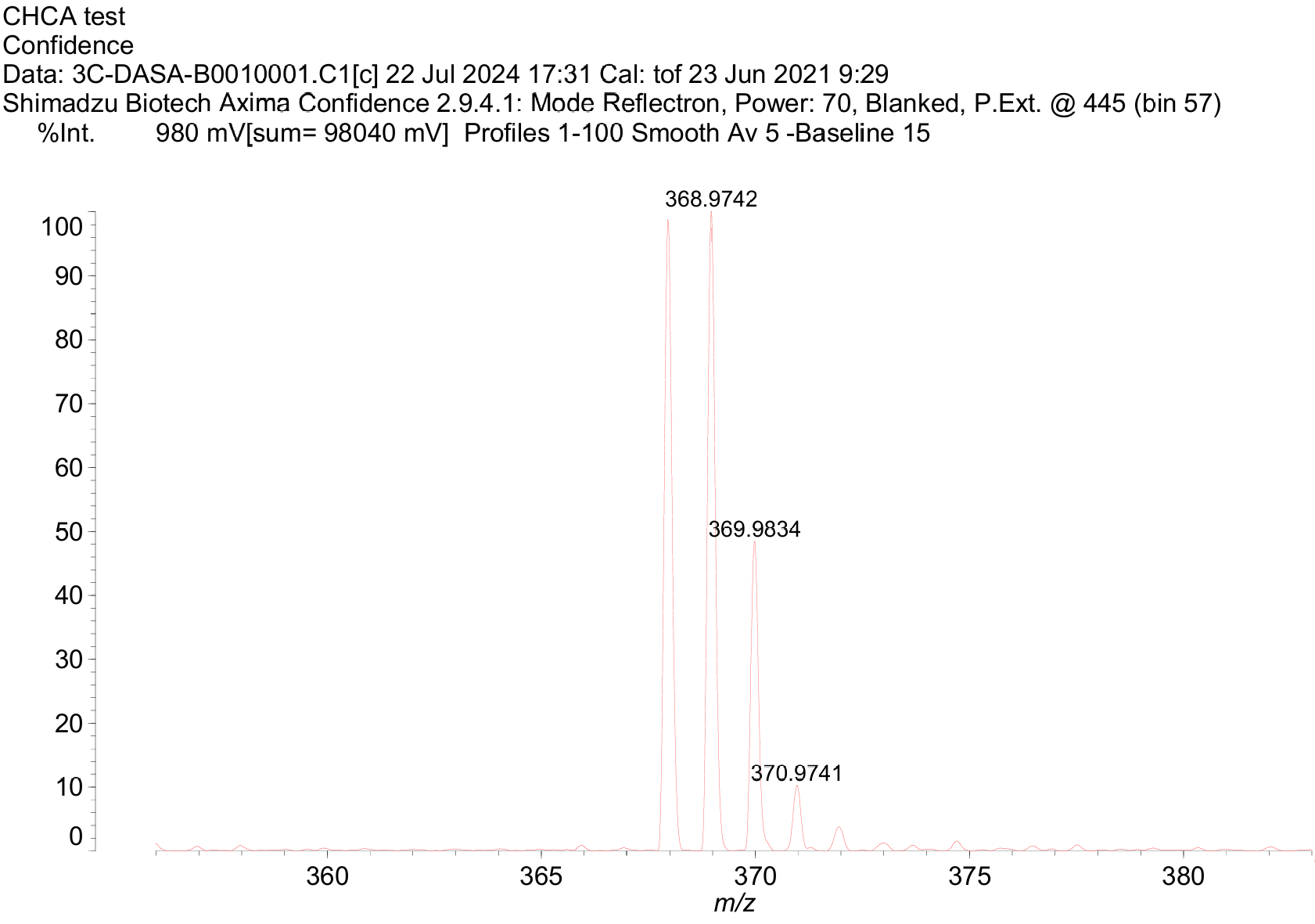


**Figure S41** High-resolution mass spectra of DASA-2.

# Supplementary movies

**Movie S1.** Video of “left-to-right” dynamic display of the fluorescent snowflake under 420 nm light irradiation (20 mW/cm^2^).

**Movie S2.** Video of “sides-to-middle” dynamic display of the fluorescent snowflake under 420 nm light irradiation (20 mW/cm^2^).

**Movie S3.** Video of “inside-to-outside” dynamic display of the fluorescent snowflake under 420 nm light irradiation (20 mW/cm^2^).

**Movie S4.** Video of “outside-to-inside” dynamic display of the fluorescent snowflake under 420 nm light irradiation (20 mW/cm^2^).

**Movie S5.** Video of the numeric pattern under 420 nm light irradiation (20 mW/cm^2^), showing sequential emergence of “134” and “888”.

**Movie S6.** Video of the symbolic pattern under 420 nm light irradiation (20 mW/cm^2^), showing sequential emergence of “China” in English and Chinese.

# Source code

Source code: self-written brightness-analysis software

function interactiveThresholdAdjuster()

I = imread('img.png');

if size(I,3) == 3

I = rgb2gray(I);

end

threshold = 128;

f1 = figure('Name', 'Interactive Threshold Tool', 'NumberTitle', 'off', ...

'Position', [100 100 900 400]);

subplot(1,3,1);

imshow(I);

title('Original Image');

uipanel('Title','Threshold Controls','Position',[0.7 0.8 0.25 0.15]);

h = uicontrol('Style', 'slider', 'Min', 0, 'Max', 255, 'Value', threshold, ...

'Position', [650 350 200 20], ...

'SliderStep', [1/255 10/255], ...

'Callback', @updateThreshold);

uicontrol('Style', 'text', 'Position', [650 320 80 20], ...

'String', 'Threshold:');

thresholdText = uicontrol('Style', 'edit', 'Position', [730 320 50 20], ...

'String', num2str(threshold), ...

'Callback', @editCallback);

uicontrol('Style', 'pushbutton', 'String', '+1', 'Position', [790 320 30 20], ...

'Callback', @(src,~) adjustThreshold(1));

uicontrol('Style', 'pushbutton', 'String', '-1', 'Position', [830 320 30 20], ...

'Callback', @(src,~) adjustThreshold(-1));

subplot(1,3,2);

maskHandle = imshow(I > threshold);

title(['Binary Mask (Threshold = ' num2str(threshold) ')']);

subplot(1,3,3);

result = I;

result(I <= threshold) = 0;

resultHandle = imshow(result);

title('Thresholded Image');

function updateThreshold(src, ~)

threshold = round(get(src, 'Value'));

updateDisplay();

end

function editCallback(src, ~)

newVal = str2double(get(src, 'String'));

if ~isnan(newVal) && newVal >= 0 && newVal <= 255

threshold = round(newVal);

updateDisplay();v

else

set(src, 'String', num2str(threshold));

end

end

function adjustThreshold(delta)

threshold = min(max(threshold + delta, 0), 255);

updateDisplay();

end

function updateDisplay()

set(h, 'Value', threshold);

set(thresholdText, 'String', num2str(threshold));

mask = I > threshold;

set(maskHandle, 'CData', mask);

title(subplot(1,3,2), ['Binary Mask (Threshold = ' num2str(threshold) ')']);

result = I;

result(~mask) = 0;

set(resultHandle, 'CData', result);

end

end

# Supporting References

[1] a) J. R. Hemmer, S. O. Poelma, N. Treat, Z. A. Page, N. D. Dolinski, Y. J. Diaz, W. Tomlinson, K. D. Clark, J. P. Hooper, C. Hawker, J. Read de Alaniz, *Journal of the American Chemical Society* **2016**, 138, 13960; b) M. M. Sroda, F. Stricker, J. A. Peterson, A. Bernal, J. Read de Alaniz, *Chemistry – A European Journal* **2021**, 27, 4183; c) F. Sun, X. Xiong, A. Gao, Y. Duan, L. Mao, L. Gu, Z. Wang, C. He, X. Deng, Y. Zheng, D. Wang, *Chemical Engineering Journal* **2022**, 427, 132037.
